# A Spatiotemporal Single‐Cell Atlas Uncovers Dysregulated ECM Dynamics and Septal Remodeling Arrest in Human Ventricular Septal Defects

**DOI:** 10.1002/advs.78016

**Published:** 2026-09-27

**Authors:** Xiaoyuan Zhang, Lun Zhu, Ze Sun, Ruolin Jia, Ziming Wang, Jiayao Zhao, Bohao Liu, Haiqing Xiong, Xiaowei Yu, Yanzhu Yue

**Affiliations:** ^1^ Prenatal Diagnosis Center Reproductive Medicine Center Department of Cell Fate and Diseases Jilin Provincial Key Laboratory of Women's Reproductive Health Jilin Provincial Clinical Research Center for Birth Defect and Rare Disease The First Hospital of Jilin University Changchun Jilin China; ^2^ Prenatal Diagnosis Center Reproductive Medicine Center the First Hospital of Jilin University Changchun Jilin China; ^3^ State Key Laboratory of Experimental Hematology National Clinical Research Center for Blood Diseases Haihe Laboratory of Cell Ecosystem Institute of Hematology & Blood Diseases Hospital Chinese Academy of Medical Sciences & Peking Union Medical College Tianjin China; ^4^ Tianjin Institutes of Health Science Tianjin China

**Keywords:** extracellular matrix remodeling, heart development, microenvironmental insufficiency, MMP2 (matrix metalloproteinase 2), ventricular septal defect

## Abstract

Precise extracellular matrix (ECM) coordination is essential for cardiac septation, yet the molecular etiology of human isolated perimembranous ventricular septal defects (VSD) remains elusive. Here, we constructed a high‐resolution single‐cell transcriptomic atlas of human VSD, benchmarking the pathological state against a 7–22 week developmental trajectory. Integrating these single‐cell profiles with a reference‐based spatial transcriptomic map, we deconvolved the distinct molecular signatures of membranous and muscular septal regions. We found that aberrant cardiomyocyte hypertrophy operates alongside a systemic failure in the non‐myocyte microenvironment. Crucially, the ECM regulatory network is dysregulated, driven by attenuated endothelial‐fibroblast crosstalk and downregulated THBS1‐integrin signaling. Supported by in vitro assays, this dysregulation impairs key endothelial‐to‐mesenchymal transition (EndoMT) programs and transcriptionally suppresses MMP2. Spatial reconstruction confirmed this maturation block compromises both septal regions, indicating a global remodeling arrest that precludes physical closure. Validated by in situ immunofluorescence and a robust correlation between reduced maternal serum MMP2 levels and defect size, our study portrays VSD as a disease driven by microenvironmental insufficiency and arrested remodeling, providing an integrative framework for understanding human cardiac dysmorphogenesis.

## Graphical abstract

Integrating single‐cell and spatial transcriptomics reveals human ventricular septal defect (VSD) as a disease of microenvironmental insufficiency and structural remodeling arrest. Attenuated endothelial‐fibroblast crosstalk, alongside downregulated THBS1/TGF‐β1 and MMP2 signaling networks, impairs the endothelial‐to‐mesenchymal transition (EndoMT) and precludes physical septal closure.

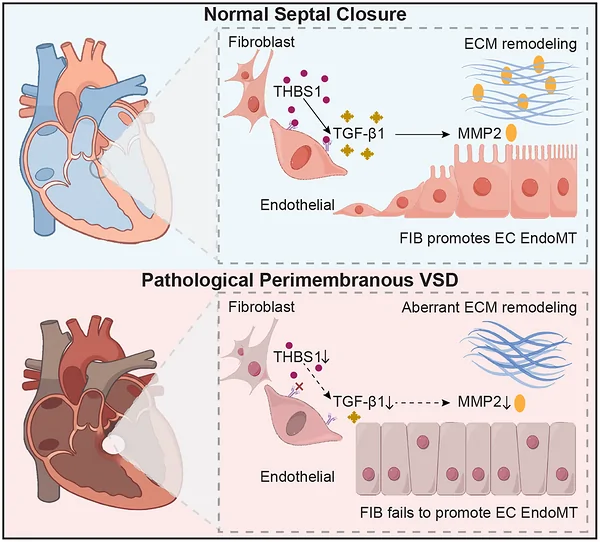

**Abbreviations**
α‐SMA: Alpha smooth muscle actin
ACM: Atrial cardiomyocyte
AV cushion: Atrioventricular cushion
ATP: Adenosine 5'‐triphosphate
BC: Blood cell
BMP: Bone morphogenetic protein
CHD: Congenital heart disease / Congenital heart defect
CI: Confidence interval
CM: Cardiomyocyte
CME: Extracellular matrix‐enriched cardiomyocyte
CSA: Cross‐sectional area
DEG: Differentially expressed gene
EC: Endothelial cell
ECM: Extracellular matrix
EDC: Endocardial cell
ELISA: Enzyme‐linked immunosorbent assay
EndoMT: Endothelial‐to‐mesenchymal transition
EMT: Epithelial‐to‐mesenchymal transition
EPDC: Epicardium‐derived cell
EPI: Epicardial cell
FDR: False discovery rate
FIB: Fibroblast
GO: Gene ontology
GSEA: Gene set enrichment analysis
HCF: Human cardiac fibroblast
HCMEC: Human cardiac microvascular endothelial cell
H&E: Hematoxylin and eosin
IF: Immunofluorescence
IVS: Interventricular septum
LTBP1/LTBP4: Latent transforming growth factor beta binding protein 1 / 4
LV/RV: Left ventricle / Right ventricle
MMP2: Matrix metallopeptidase 2
MP: Macrophage
NC: Neural crest‐derived cell
NKX2‐5: NK2 homeobox 5
NPPA/NPPB: Natriuretic peptide A / B
PC: Proliferating cell
PCA: Principal component analysis
PCW: Post‐conception week
PPIA: Peptidylprolyl isomerase A (Cyclophilin A)
Pro_CM: Proliferative cardiomyocyte
RA: Right atrium
scRNA‐seq: Single‐cell RNA sequencing
SMC: Smooth muscle cell
ST: Spatial transcriptomics
TGF‐β1: Transforming growth factor‐beta‐1
THBS1: Thrombospondin 1
UMAP: Uniform manifold approximation and projection
VCM: Ventricular cardiomyocyte
VSD: Ventricular septal defect
WGA: Wheat germ agglutinin

## 1 Introduction

Substantial morphological remodeling during early cardiac development—from linear tube formation to septation—ensures the establishment of unidirectional circulation and structural integrity. In humans, perturbations in these highly orchestrated morphogenic events lead to congenital heart defects, among which perimembranous ventricular septal defect (VSD) is the most prevalent [1]. The ventricular septum comprises distinct muscular and membranous components, originating from the expansion and coalescence of trabeculated myocytes and the subsequent closure of the embryonic interventricular communication [2, 3, 4]. Following the connection of the left ventricle to the subaortic outlet via the secondary foramen, the muscular crest aligns with the proximal outflow cushions, which subsequently myocardialise to form the subpulmonary infundibulum. This process leaves a residual opening—the tertiary interventricular foramen—that is ultimately closed by the tubercles of the atrioventricular cushions. Subsequent delamination of the tricuspid septal leaflet divides the membranous septum into its atrioventricular and interventricular components [4, 5, 6]. Crucially, failure to close this tertiary interventricular foramen results in a perimembranous ventricular septal defect (VSD), the most prevalent congenital heart anomaly. Despite its frequency, the precise cellular movements and local signals driving this specific closure in human embryos remain critically underexplored.

Mapping the spatiotemporal transcriptomic profiles that cover cellular lineages in the pathological state is a fundamental goal in cardiovascular medicine. However, this effort has been hindered by the scarcity of high‐fidelity human VSD specimens and the difficulty in decoupling primary morphogenic defects from secondary hemodynamic adaptation [7, 8, 9]. Current insights into septal formation are largely derived from murine models or healthy human atlases. While these studies have characterized healthy development, the specific pathological alterations in cellular composition, endothelial‐to‐mesenchymal transition (EndoMT), and ECM remodeling within the context of human isolated perimembranous VSD remain a critical knowledge gap.

Further dissection of these underlying molecular mechanisms remains challenging owing to the intricate anatomical heterogeneity of the human heart, where the membranous and muscular septa exhibit distinct developmental vulnerabilities. Here, we address these barriers by constructing a single‐cell atlas of human isolated perimembranous VSD, leveraging reference‐based spatial integration to reconstruct the anatomical architecture of the pathological septum. By projecting rare pathological transcriptomes onto a continuous healthy developmental continuum (7–22 weeks), we systematically decoded the cellular heterogeneity of the septum, revealing a complex pathological state defined by metabolic stress in cardiomyocytes and functional dysregulation in the stromal niche. Through cellular interaction analysis and subsequent in vitro functional perturbation assays, we further identify a specific bottleneck in the crosstalk‐remodeling axis, the attenuation of the THBS1–TGF‐β1 signaling axis, the impairment of EndoMT, and the subsequent suppression of *MMP2*. Finally, we mapped this molecular deficit to the anatomical architecture of the septum, revealing a global structural maturation arrest that compromises both membranous and muscular regions. Collectively, this work establishes a spatially resolved molecular blueprint for the failing septum, redefining isolated perimembranous VSD as a developmental disorder fundamentally driven by microenvironmental insufficiency and arrested tissue remodeling.

## 2 Results

### 2.1 Single‐Cell Transcriptomic Atlas of Human Cardiac Development and VSD Pathology

**FIGURE 1:**
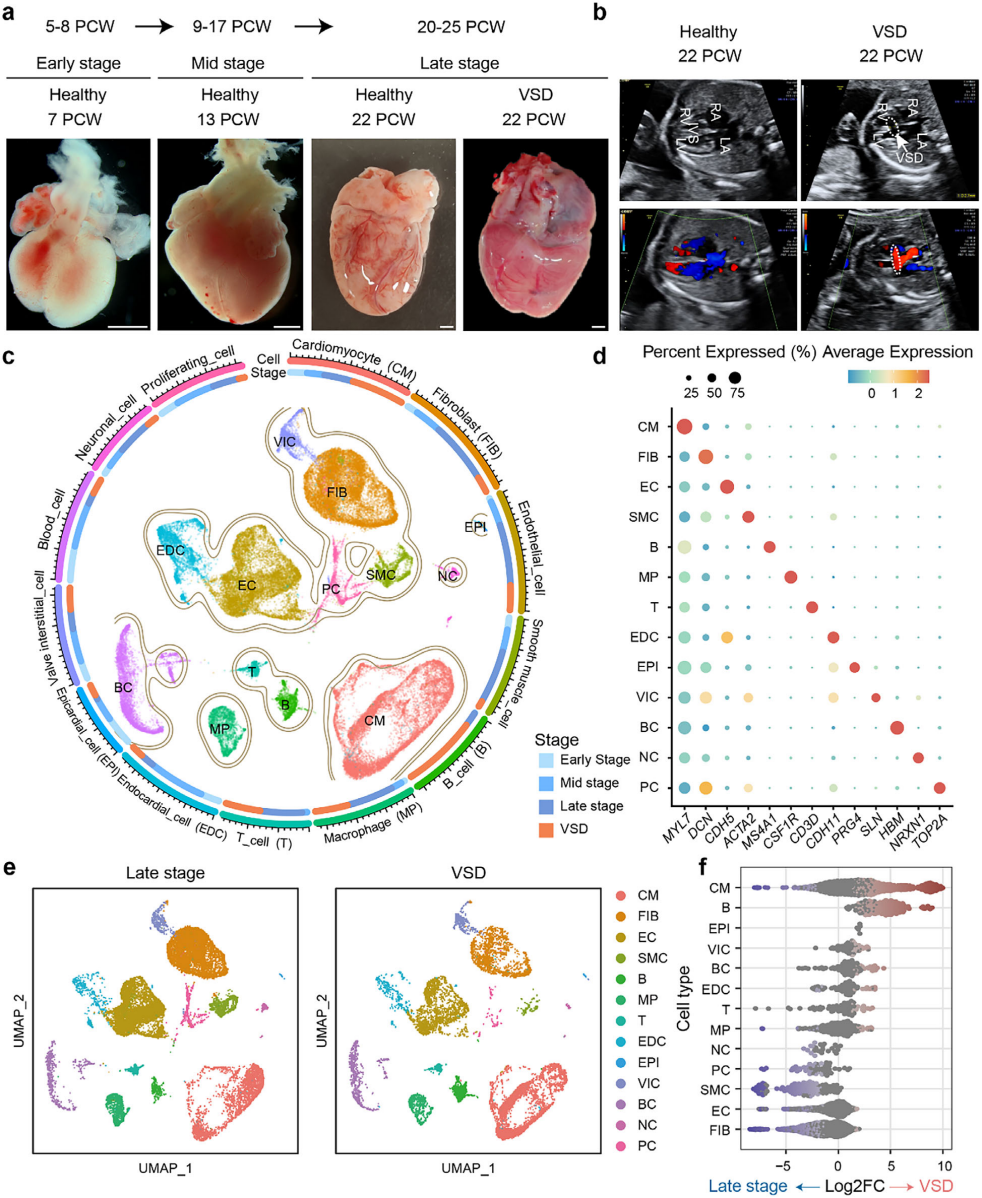
Single‐cell transcriptomic atlas of human cardiac development and VSD pathology. (a) Gross morphology of human fetal hearts across three developmental stages (Early, 5–8 PCW; Mid, 9–17 PCW; Late, 20–25 PCW) compared with Late stage VSD hearts. Scale bars, 2 mm. (b) Echocardiographic assessment of healthy versus VSD hearts at 22 weeks of gestation. Top panels show B‐mode parasternal long‐axis views; bottom show Color Doppler imaging revealing turbulent left‑to‑right shunt flow (arrowheads) across the ventricular septum. RA, right atrium; RV, right ventricle; LA, left atrium; LV, left ventricle; IVS, interventricular septum. (c) UMAP embedding of the integrated single‐cell dataset (*n* = 53 410 cells) derived from developing human hearts across early, mid, and Late stages and VSD patients. Cells are annotated into 13 major cell types. Concentric tracks denote cluster identity (outer) and stage‐specific contribution (inner). (d) Dot plots characterizing the expression of canonical cell type markers. Dot size encodes the fraction of expressing cells and color gradient represents mean scaled expression levels. (e) Comparative UMAP manifolds of Late stage healthy (left, *n* = 19 903) and VSD (right, *n* = 13 380) hearts, colored by cell type. (f) Distribution of log2 fold change (Log2FC) values for cell type abundance in VSD samples relative to Late stage controls. Positive values indicating enrichment in VSD, while negative values indicate depletion.

To systematically characterize the cellular and molecular landscapes underlying human cardiac morphogenesis and septation failure, we constructed a single‐cell transcriptomic atlas of human fetal heart tissues spanning three critical developmental stages: early (5–8 weeks), mid (9–17 weeks), and late (20–25 weeks) gestation (Table S1). Additionally, to investigate the pathological alterations associated with congenital defects, we collected two stage‐matched fetal heart samples (22 weeks) that were diagnosed with isolated perimembranous ventricular septal defects (VSDs). Gross morphological assessment confirmed that while normal hearts exhibited progressive structural maturation, VSD hearts displayed marked ventricular and atrial hypertrophy (Figure 1a). This phenotype was further validated by B‐mode echocardiography and Color Doppler imaging, which revealed a distinct septal discontinuity and turbulent left‑to‑right shunt flow (Figure 1b). These findings confirmed the anatomical lesion and suggested a secondary remodeling response to hemodynamic stress in VSD hearts.

We subsequently constructed a comprehensive single‑cell atlas comprising 53 410 high‑quality single cells from eight whole‐heart samples (six healthy controls across developmental stages and two VSD cases) (Figure S1a,b). Correlation analysis confirmed high reproducibility between hearts from different embryos at the same developmental stage, and integrated analysis demonstrated the effective removal of batch effects, with cells from different samples mixing well within clusters (Figure S1c,d). Additionally, both the developmental progression of the samples and the pathological divergence of VSD samples from healthy controls were accurately captured (Figure S1e). Unsupervised clustering identified 13 major cell types based on the expression of canonical marker genes, including cardiomyocytes (CM; *MYL7*), fibroblasts (FIB; *DCN*), endothelial cells (EC; *CDH5*), smooth muscle cells (SMC; *ACTA2*), B cells (*MS4A1*), macrophages (MP; *CSF1R*), T cells (*CD3D*), endocardial cells (EDC; *CDH11*), epicardial cells (EPI; *PRG4*), valvular interstitial cells (VIC; *SLN*), blood cells (BC; *HBM*), neural crest‑derived cells (NC; *NRXN1*), and proliferating cells (PC; *TOP2A*) (Figure 1c,d).

Comparative analysis of cellular composition revealed profound remodeling of the cardiac ecosystem in VSD (Figure 1e,f). Specifically, the CM and B cell populations were markedly expanded in VSD samples compared to Late stage controls, consistent with the observed hypertrophy and potential inflammatory involvement. In contrast, non‐myocyte stromal lineages including FIB, EC and SMC were notably decreased. The alterations in cell type proportions in VSD patients observed in our scRNA‐seq data were further quantified and validated by immunofluorescence (IF) at the tissue level (Figure S2). This significant reduction in structural and vascular support cells suggests that perimembranous VSD pathology might be associated with a dysregulation of the non‐myocyte microenvironment, prompting us to further investigate the functional alterations within these lineages.

### 2.2 Deciphering the Developmental Trajectory of Cardiomyocytes and Their Dysregulation in VSD

**FIGURE 2:**
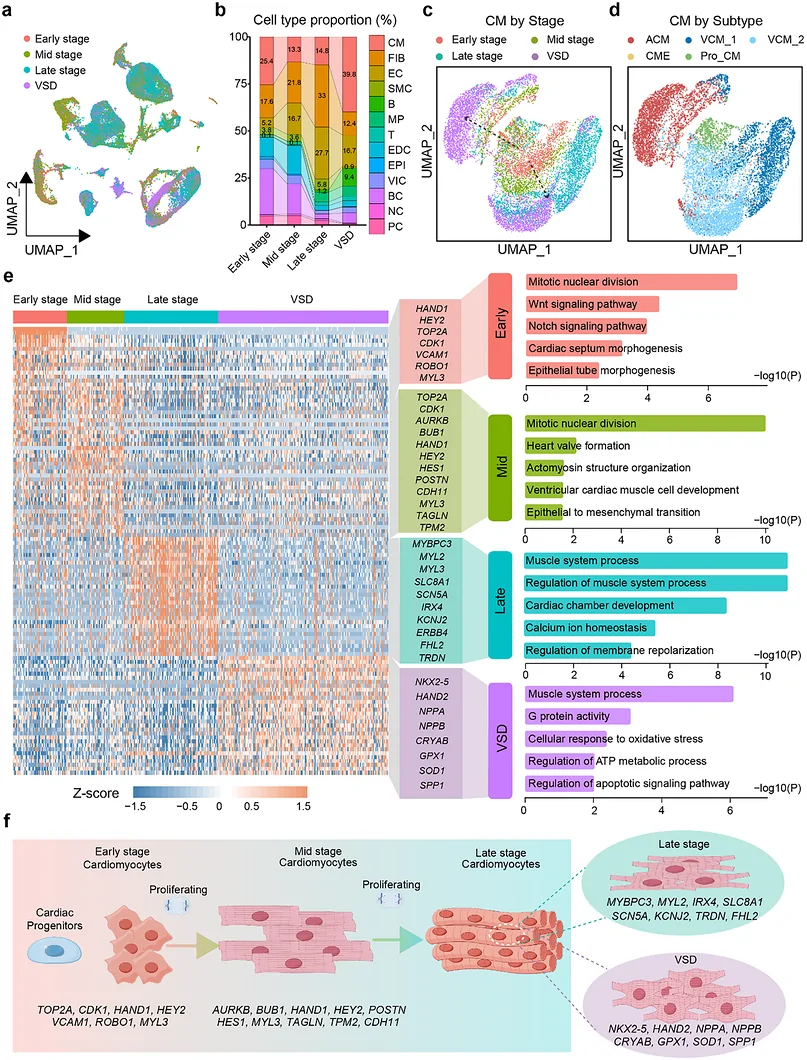
Transcriptional trajectory of cardiomyocyte development and stress‐induced maladaptation in VSD. (a) Global UMAP projection of the integrated dataset, colored by sample identity to visualize the distribution of cells from early, mid, and late developmental stages and VSD samples. (b) Relative abundance of major cell lineages across the four sample groups, visualized by stacked bar charts showing the proportional contribution of each cell type. (c, d) Sub‐clustering analysis of the cardiomyocyte (CM) lineage. UMAP manifolds are colored by sample group (c) and annotated subtype (d). ACM, atrial cardiomyocytes; VCM_1/2, ventricular CM subtypes; CME, ECM‐enriched CM; Pro_CM, proliferative CM. (e) Heatmap showing stage‐specific gene expression signatures. Rows represent Z‐score scaled expression of marker genes (left), with representative genes highlighted (middle). Bar plots (right) show the significance (‐log10 *p*‐value) of enriched Gene Ontology (GO) biological processes for each gene module. Note that VSD cardiomyocytes are characterized by stress response and metabolic pathways rather than developmental arrest. (f) Schematic model illustrating the physiological maturation trajectory of cardiomyocytes—from proliferation to structural specialization—contrasted with the VSD pathology, which exhibits secondary adaptive responses including oxidative stress and metabolic dysregulation.

Given the apparent expansion of the myocardial compartment observed in VSD hearts, we prioritized the CM lineage for high‐resolution analysis to determine the nature of this accumulation (Figure 1). Dimensionality reduction via UMAP resolved the integrated dataset into distinct clusters corresponding to major lineages, confirming the capture of a continuous developmental trajectory from early gestation to the late fetal stage (Figure 2a). Analysis of cellular composition revealed dynamic shifts during physiological development, characterized by a progressive decrease in the relative proportion of CMs, concomitant with an expansion of non‐myocyte stromal populations, including FIBs and ECs [10]. In VSD hearts, however, this physiological transition was disrupted. VSD samples retained a high proportion of CMs comparable to early developmental stages, yet exhibited a concurrent depletion of stromal support cells (Figure 2b).

To dissect the intrinsic heterogeneity associated with this phenotype, we performed sub‐clustering of the cardiomyocyte lineage (Figure S3). Trajectory reconstruction delineated a continuous physiological continuum, spanning from early proliferative progenitors to terminally differentiated states (Figure 2c). We identified five distinct subpopulations: proliferative CM (Pro_CM), extracellular matrix‐enriched CM (CME), atrial CM (ACM), and two ventricular subsets (VCM 1/2) (Figure 2d). Notably, the trajectory analysis confirmed that atrial and ventricular lineages diverge shortly after the proliferative progenitor stage, establishing the molecular foundation for chamber‐specific specialization.

We further characterized the core molecular programs driving physiological maturation by defining stage‐specific transcriptional modules. The early stage was defined by a proliferation and fate determination signature, marked by high expression of cell cycle genes (*TOP2A*, *CDK1*) and core transcription factors (*HAND1*, *IRX4*). As development progressed to the mid stage, cells transitioned into a morphogenesis and septation phase, characterized by the upregulation of tissue remodeling genes (*POSTN*, *CDH11*) and the actomyosin organization pathway. By the Late stage, cardiomyocytes achieved functional maturation, distinguishing themselves by the robust expression of genes regulating the contractile apparatus (*MYBPC3*, *TPM2*) and calcium handling regulators (*SLC8A1*, *SCN5A*) (Figure 2e).

Projection of VSD cardiomyocytes onto this developmental trajectory revealed a distinct pathological deviation. While VSD samples predominantly clustered with Late stage controls (Figure 2c), they displayed a specific hypertrophic stress module [11] (Figure 2e). Unlike normal late‐stage cells, VSD cardiomyocytes significantly upregulated markers of pathological hypertrophy (*NPPA*, *NPPB*) alongside genes associated with the cellular response to oxidative stress (*CRYAB*, *SOD1*) and macrophage cytokine production (*SPP1*) (Figure 2e). These findings suggest that VSD cardiomyocytes, while structurally differentiated, are diverted into a functionally compromised state characterized by metabolic stress and hypertrophic signaling, distinct from the physiological maturation observed in healthy controls (Figure 2f).

### 2.3 Lineage‐Specific Hypertrophy and Divergent Differentiation Trajectories in VSD

**FIGURE 3:**
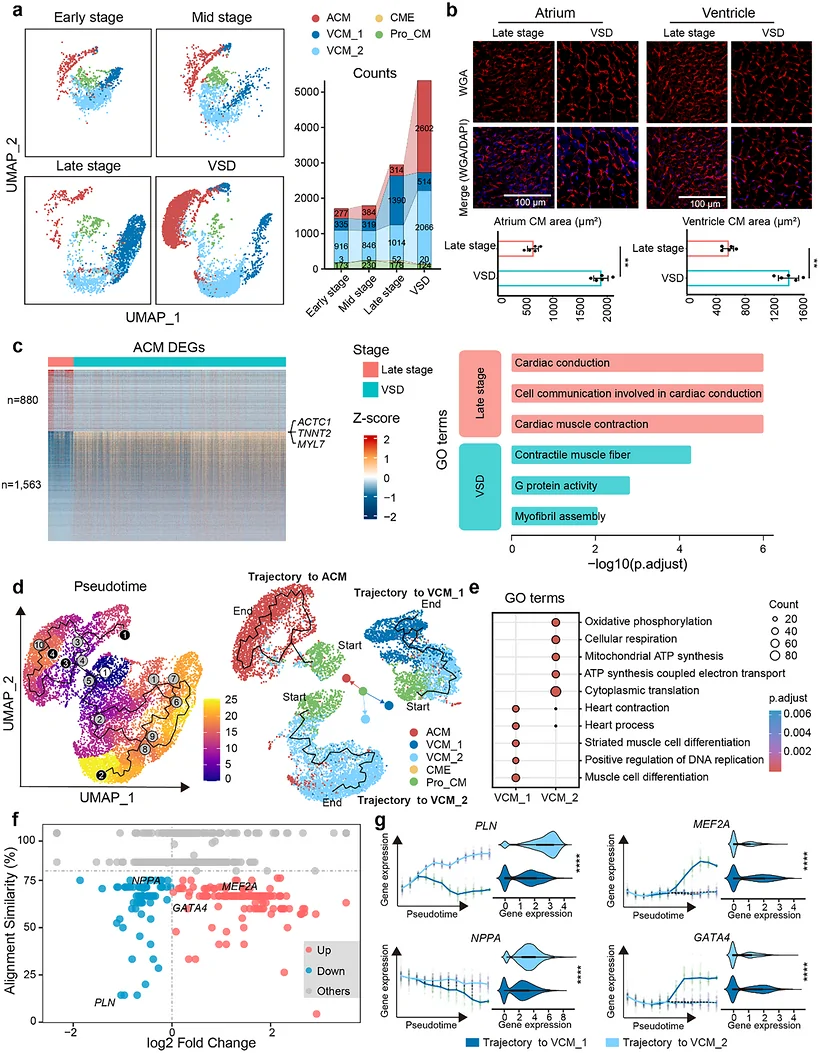
Lineage‐specific hypertrophy and divergent differentiation trajectories in VSD. (a) UMAP projection of the re‐clustered cardiomyocyte lineage, revealing distinct Atrial (ACM) and Ventricular (VCM) subpopulations across developmental stages and VSD samples. Stacked bar plots (right) show the absolute cell counts per stage. (b) Representative Wheat Germ Agglutinin (WGA) staining of atrial and ventricular myocardium, demonstrating significant cellular hypertrophy in VSD samples compared with Late stage controls. Quantification of cardiomyocyte cross‐sectional area (CSA) is shown on the right (*n* = 2 biological replicates per group, with 5 randomly selected microscopic fields per group; Data are presented as mean ± 95% CI; ***P < 0.001, by two‐sided *Wilcoxon tests*). Scale bars, 100 µm. (c) Transcriptional signature of remodeling in atrial cardiomyocytes (ACM). Heatmap (left) displaying stage‐specific differentially expressed genes (DEGs) within the ACM lineage (red indicates high expression; blue indicates low), alongside bar plots (right) quantifying enriched GO biological processes (bar height represents ‐log10 *P*‐value). (d) Pseudotime trajectory analysis tracing the differentiation lineage from proliferative progenitors (Pro_CM) into divergent VCM_1 and VCM_2 fates, colored by pseudotime values. (e) Functional characterization of ventricular subtypes using dot plots to visualize distinct biological processes distinguishing VCM_1 and VCM_2 populations. Dot size represents gene count, and color indicates adjusted *P‐*value. (f) Alignment similarity analysis (top) identifying driver genes of ventricular bifurcation. Genes are plotted against log2 fold change of mean expression, highlighting upregulation (red) or downregulation (blue) in VSD. (g) Temporal expression dynamics of selected transcription factors (e.g., *MEF2A* and *GATA4*) and functional markers (*PLN* and *NPPA*) along the pseudotime axis from VCM_1 (*n* = 3586) and VCM_2 (*n* = 4299) trajectories. ****P < 0.0001 by two‐sided *Wilcoxon tests*.

To dissect the lineage‐specific alterations associated with this pathology, we first visualized the sub‐clustered cardiomyocyte landscape. UMAP projection clearly delineated the two major lineages, ACM and VCM, revealing a significant remodeling of VCM subpopulation proportions in VSD hearts compared to controls (Figure 3a). To determine whether this transcriptomic shift translates into a physical phenotype, we assessed cellular morphology in situ. Wheat Germ Agglutinin (WGA) staining demonstrated a significant increase in the cross‐sectional area (CSA) of cardiomyocytes in VSD samples. This cellular hypertrophy was lineage‐inclusive, affecting both the atrial and ventricular myocardium (Figure 3b). In the ACM lineage, this was molecularly corroborated by the significant upregulation of sarcomere and myofibrillar structural genes (*ACTC1*, *TNNT2*, and *MYL7*) in VSD, which were functionally enriched in contractile muscle fiber pathways (Figure 3c), suggesting a direct adaptive response to hemodynamic overload.

Within the ventricular lineage, pseudotime trajectory analysis revealed a profound bifurcation in cell fate decisions. We reconstructed the differentiation path from Pro_CM progenitors, which branched into two distinct termini: VCM_1 and VCM_2 (Figure 3d). Functional enrichment analysis defined this divergence: The VCM_1 branch represented the canonical maturation trajectory, enriched for muscle cell differentiation and heart contraction, reflecting the acquisition of normal contractile competence. In contrast, the VCM_2 branch, which was preferentially enriched in VSD hearts, was dominated by metabolic and stress‐related pathways, including oxidative phosphorylation and mitochondrial ATP synthesis (Figure 3e).

This trajectory bifurcation suggests a model in which the VSD microenvironment alters normal ventricular differentiation, correlating with a shift in cell fate toward a compensatory stress state (VCM_2). To elucidate the transcriptional dynamics underlying this deviation, we analyzed gene expression dynamics along the pseudotime axis (Figure 3f,g). Along the VCM_1 trajectory, key transcription factors such as *MEF2A* and *GATA4* were steadily upregulated in synchrony with maturation. Conversely, the VCM_2 trajectory exhibited a dysregulated pattern: the expression of the maturation factor *MEF2A* lagged, while hypertrophy markers (*NPPA*) and calcium regulatory genes (*PLN*) were abnormally elevated. Collectively, these data suggest that VSD is associated with an alteration of the canonical ventricular differentiation program, coinciding with a cellular transition toward a compensatory, metabolically demanding hypertrophic state.

### 2.4 Altered ECM Dynamics and Identification of a Preliminary, Non‐Invasive Biomarkers in VSD

**FIGURE 4:**
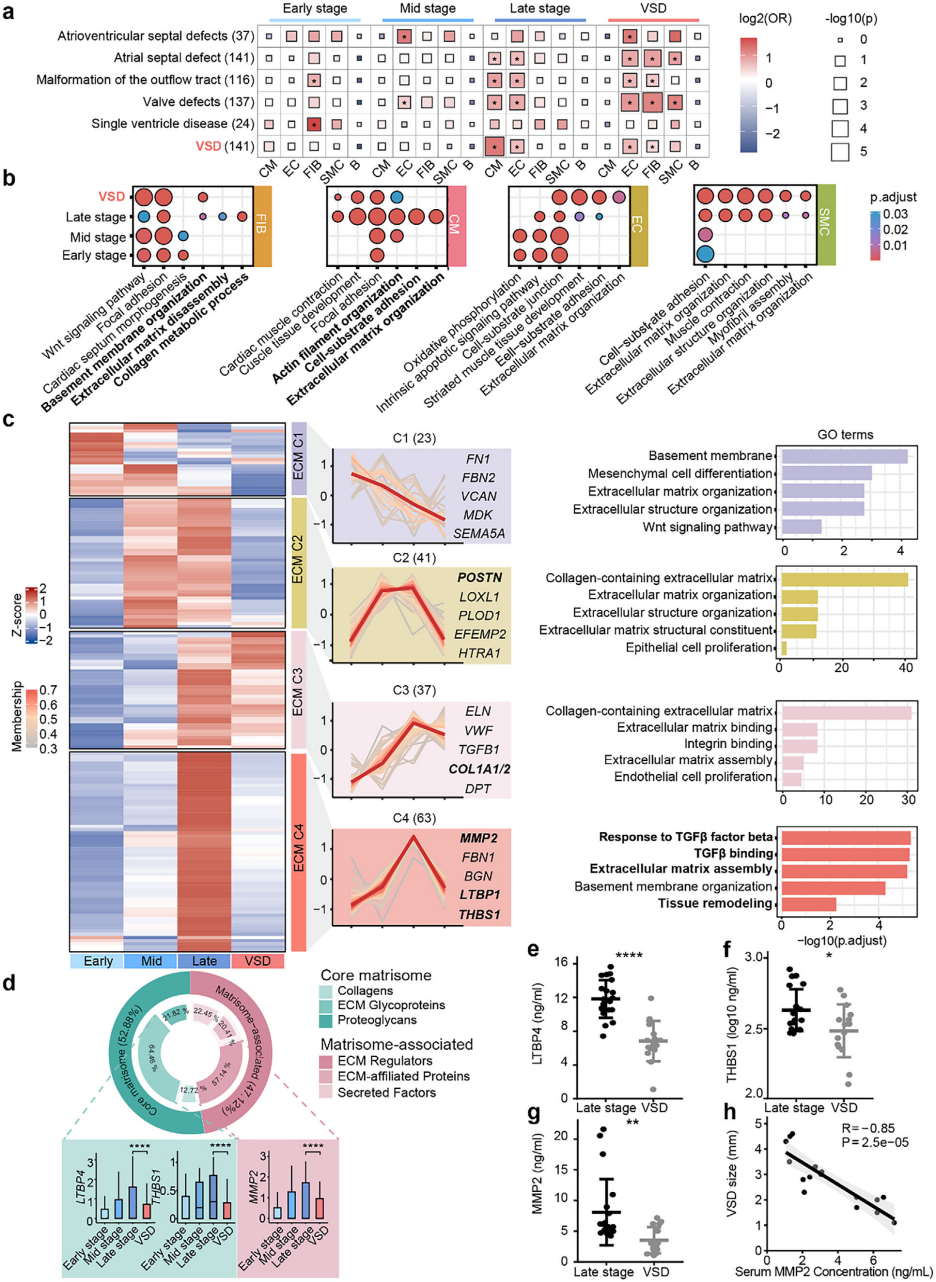
Altered ECM landscape and identification of non‐invasive biomarkers for VSD. (a) Heatmap displaying the statistical significance of overlap between cell‐type specific signatures and known cardiovascular risk loci, calculated by a one‐sided Fisher's exact test (*P < 0.05). (b) Temporal functional profiling of five major cell types. Dot plots illustrate the enrichment of ECM‐related GO biological processes, with dot size representing gene count and color intensity representing significance. (c) Expression dynamics of the cardiac matrisome (MatrisomeDB 2.0), analyzed by clustering ECM genes into four distinct modules (C1–C4). The panel shows a heatmap of expression patterns (left), line plots tracking temporal trends (middle)—notably revealing VSD‐specific downregulation in Cluster 4—and bar plots quantifying associated biological functions (right). (d) Profiling of divergent ECM constituents distinguishing Cluster 2 and Cluster 4, categorizing the identified components into core matrisome (collagens, proteoglycans) and matrisome‐associated molecules. Late stage: *n* = 19 903; VSD: *n* = 13 380. ****P < 0.0001 by two‐sided *Wilcoxon tests*. (e–g) Validation of candidate biomarkers in maternal serum using ELISA. Dot plots show concentrations of LTBP4 (e), THBS1 (f), and MMP2 (g) in healthy pregnancies (*n* = 20) versus those carrying fetuses with VSD (*n* = 16). Data are presented as mean ± 95% CI; *P < 0.05, **P < 0.01, ****P < 0.0001 by two‐sided *Wilcoxon tests*. (h) Scatter plot demonstrating the significant inverse correlation between maternal serum MMP2 concentration and the fetal VSD diameter, based on 16 VSD samples (Spearman correlation analysis, R = – 0.85, *P* = 2.5e−05).

To explore potential cellular contributors to VSD pathogenesis distinct from secondary adaptations, we integrated our single‐cell atlas with a curated set of high‐confidence congenital heart disease (CHD) risk genes [12, 13, 14, 15] (Figure 4a). Enrichment analysis revealed that pathogenic CHD transcripts were disproportionately enriched in CMs, ECs, and FIBs (Figure 4a). Furthermore, quantitative analysis confirmed that fibroblasts constitute the predominant cellular source of the cardiac extracellular matrix (ECM) throughout development (Figure S5). These data suggest that non‐myocyte stromal populations are the main source of pathogenic alterations, supporting stromal niche insufficiency as the potential epicenter of the defect.

We therefore focused on the functional output of these stromal populations. Differential expression analysis identified ECM remodeling as a significantly dysregulated biological process in VSD hearts (Figure 4b). To systematically characterize the temporal regulation of this process, we reconstructed the developmental dynamics of the cardiac matrisome. Unsupervised clustering identified distinct ECM gene modules with precise spatiotemporal patterns [16] (Table S2; Figure 4c). Cluster 4, enriched for ECM organization and TGF‐β1 regulation, showed robust expression in late‐stage controls, supporting structural maturation [1, 17, 18].

Strikingly, this dynamic ECM network was fundamentally disrupted in VSD. The most characteristic alteration was the specific downregulation of Clusters 2 and 4 in VSD samples (Figure 4c). This disruption originated not from a global shift in ECM components, but from a spatiotemporal dysregulation of key regulatory molecules.

Within this cluster, we identified a significant loss of Thrombospondin‐1 (*THBS1*) and Latent Transforming Growth Factor Beta Binding Protein 4 (*LTBP4*) (Figure 4d). As key activators and anchors of TGF‐β1 signaling, their deficiency suggests a localized signaling impairment at the septal fusion front [19, 20, 21]. To determine the cellular origin of these factors, we resolved the molecular heterogeneity of the stromal compartment, identifying distinct fibroblast subtypes, including *MMP2^+^* and *COL1A2^+^* populations, which were the primary producers of these morphogens (Figure S6). The specific suppression of *THBS1* and *LTBP4* in VSD supports a model wherein a failure in instructive crosstalk disrupts septal morphogenesis.

To explore the clinical utility of these findings, we assessed whether systemic levels of these ECM regulators correlate with the defect. Analysis of maternal serum revealed significantly reduced levels of *MMP2*, *LTBP4*, and *THBS1* in pregnancies with isolated fetal VSD compared to matched healthy controls (Table S3; Figure 4e–g). Notably, maternal serum *MMP2* levels exhibited a significant negative correlation with fetal septal defect size (Figure 4h), whereas *LTBP4* and *THBS1* did not show such a correlation (Figure S7), suggesting that circulating *MMP2* may serve as a preliminary non‐invasive biomarker reflecting the severity of arrested cardiac remodeling.

### 2.5 Aberrant Endothelial‐Fibroblast Crosstalk Correlates With ECM Dysregulation and EndoMT Impairment in VSD

We next hypothesized that the observed ECM deficiency, particularly the loss of TGF‐β1 activators, compromises the endothelial‐to‐mesenchymal transition (EndoMT) required for septal closure [22, 23, 24]. To validate this, we first examined the global developmental trajectories of non‐cardiomyocyte lineages. Trajectory analysis revealed broad transcriptional shifts in VSD fibroblasts, smooth muscle cells, and endothelial cells compared to stage‐matched controls (Figure S6a–c), characterized by the downregulation of maturation‐associated gene programs (Figure S6d,e).

**FIGURE 5:**
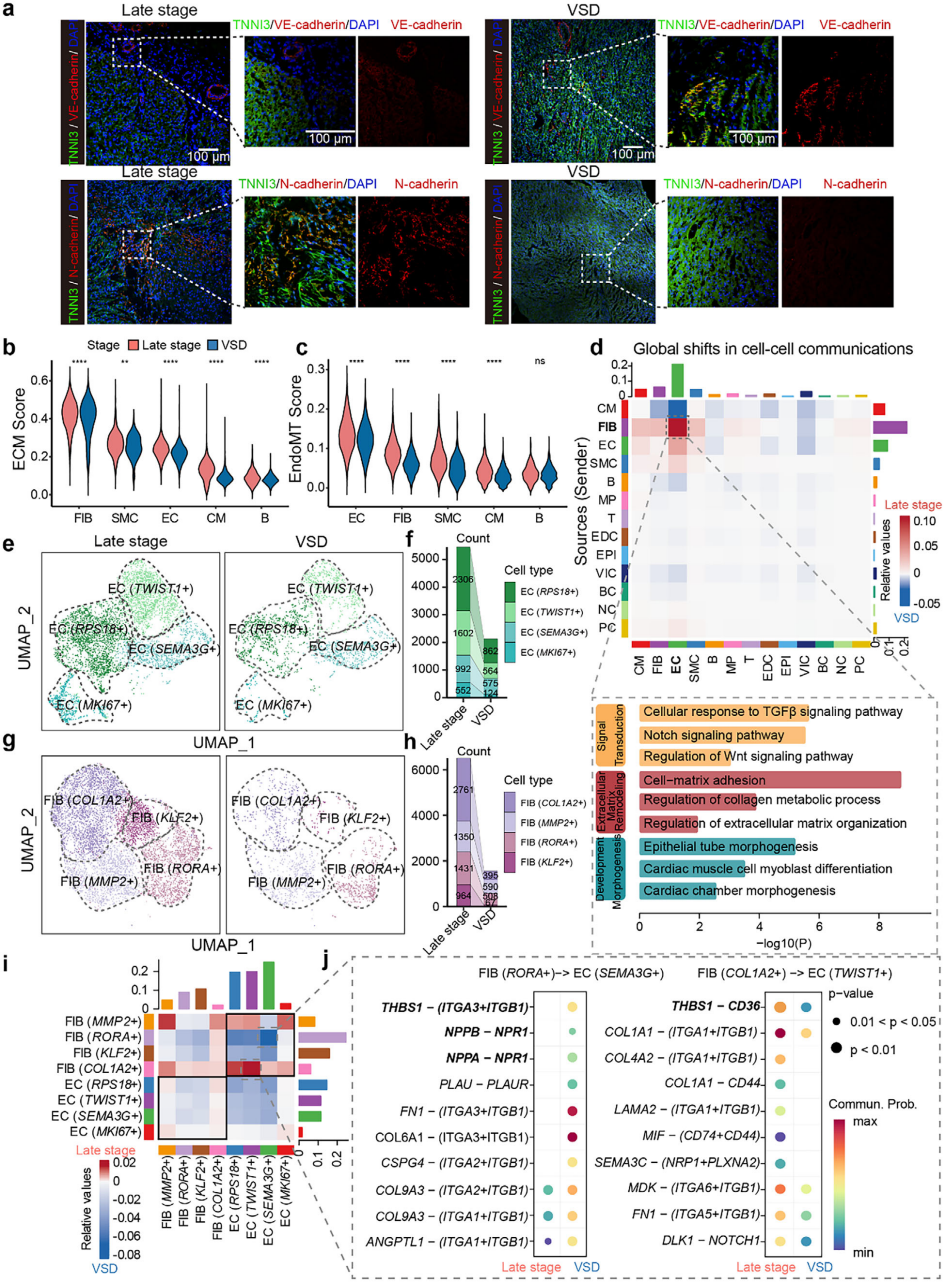
Aberrant endothelial‐fibroblast crosstalk correlates with ECM dysregulation and EndoMT in VSD. (a) Immunofluorescence visualization of cardiac tissue architecture using markers for cardiomyocytes (TNNI3), endothelial cells (VE‐cadherin), and adherens junctions (N‐cadherin), highlighting structural disorganization in VSD septa (*n* = 2) compared with Late stage controls (*n* = 2). Scale bars, 100 µm. (b, c) Violin plots quantifying pathological signatures: ECM scores (b) and EndoMT scores (c) across major cell types. ns, not significant, **P < 0.01, ****P < 0.0001 by two‐sided *Wilcoxon tests*. (d) Differential intercellular communication landscape visualized by a heatmap showing changes in interaction strength between sender (y‐axis) and receiver (x‐axis) cell populations, where red indicates increased signaling in Late‐stage and blue indicates increased signaling in VSD. (e–h) Sub‐lineage profiling of endothelial cells (EC) and fibroblasts (FIB), with UMAP manifolds (e, g) and bar charts (f, h) illustrating the distribution and proportional shifts of subtypes, including *TWIST1* ^+^ ECs and *MMP2* ^+^ FIBs. (i) Heatmap of differential interaction strength focusing on the specific crosstalk between fibroblast subtypes (senders) and endothelial subtypes (receivers). (j) Ligand‐receptor specificity analysis visualized by a dot plot identifying significantly altered communication pairs (e.g., *THBS1*–*ITGA3*) driving fibroblast‐to‐endothelial signaling. Dot size represents *P‐*value significance, and color gradient indicates communication probability.

To determine whether these transcriptional deficits correlate with an altered cell fate transition, we evaluated EndoMT status in situ. Immunofluorescence staining of VSD septal tissues revealed an aberrant upregulation of the endothelial junction protein VE‐cadherin, concomitant with a significant downregulation of the mesenchymal marker N‐cadherin relative to controls (Figure 5a). This expression profile—characterized by the retention of endothelial traits and the attenuated acquisition of mesenchymal features—suggests an impaired EndoMT at the protein level [25]. Consistently, AUCell scoring confirmed that transcriptional signatures associated with EndoMT [26] (Table S4) and ECM remodeling were significantly attenuated in VSD endothelial and fibroblast populations, respectively (Figure 5b,c).

To dissect the intercellular signaling networks potentially mediating this process, we constructed a single‐cell receptor‐ligand interaction map (Figure 5d). Quantitative comparison of differential interaction strength revealed a distinct lineage‐specific pattern: while other axes remained relatively stable, the paracrine signaling flux originating from fibroblasts and targeting endothelial cells was profoundly suppressed in VSD. High‐resolution sub‐clustering deconvoluted the endothelial lineage into a *SEMA3G^+^* subset and a *TWIST1^+^* population poised for EndoMT (Figure 5e,f). Parallel analysis of the fibroblast compartment stratified cells into *COL1A2^+^* matrix‐producing fibroblasts and a distinct *RORA^+^* regulatory subset (Figure 5g,h).

Interaction analysis pinpointed a critical rewiring of crosstalk between these subsets (Figure 5i,j). In healthy hearts, we identified a robust *THBS1‐CD36* signaling axis connecting fibroblasts (specifically the *RORA* ^+^ and *COL1A2* ^+^ subsets) to endothelial cells. This pathway is known to regulate microvascular remodeling and EndoMT [27, 28]. In VSD, this protective axis was severely attenuated. Instead, *THBS1* signaling shifted toward the *ITGA1* integrin receptor, while the pathological *NPPA/NPPB‐NPR1* stress signaling axis was aberrantly activated. These findings suggest that the loss of instructional *THBS1‐CD36* crosstalk drives EndoMT attenuation, while compensatory stress signaling dominates the microenvironment.

### 2.6 Functional Perturbation Supports the THBS1–TGF‐β1 Axis in Mediating EndoMT and Matrix Remodeling

**FIGURE 6:**
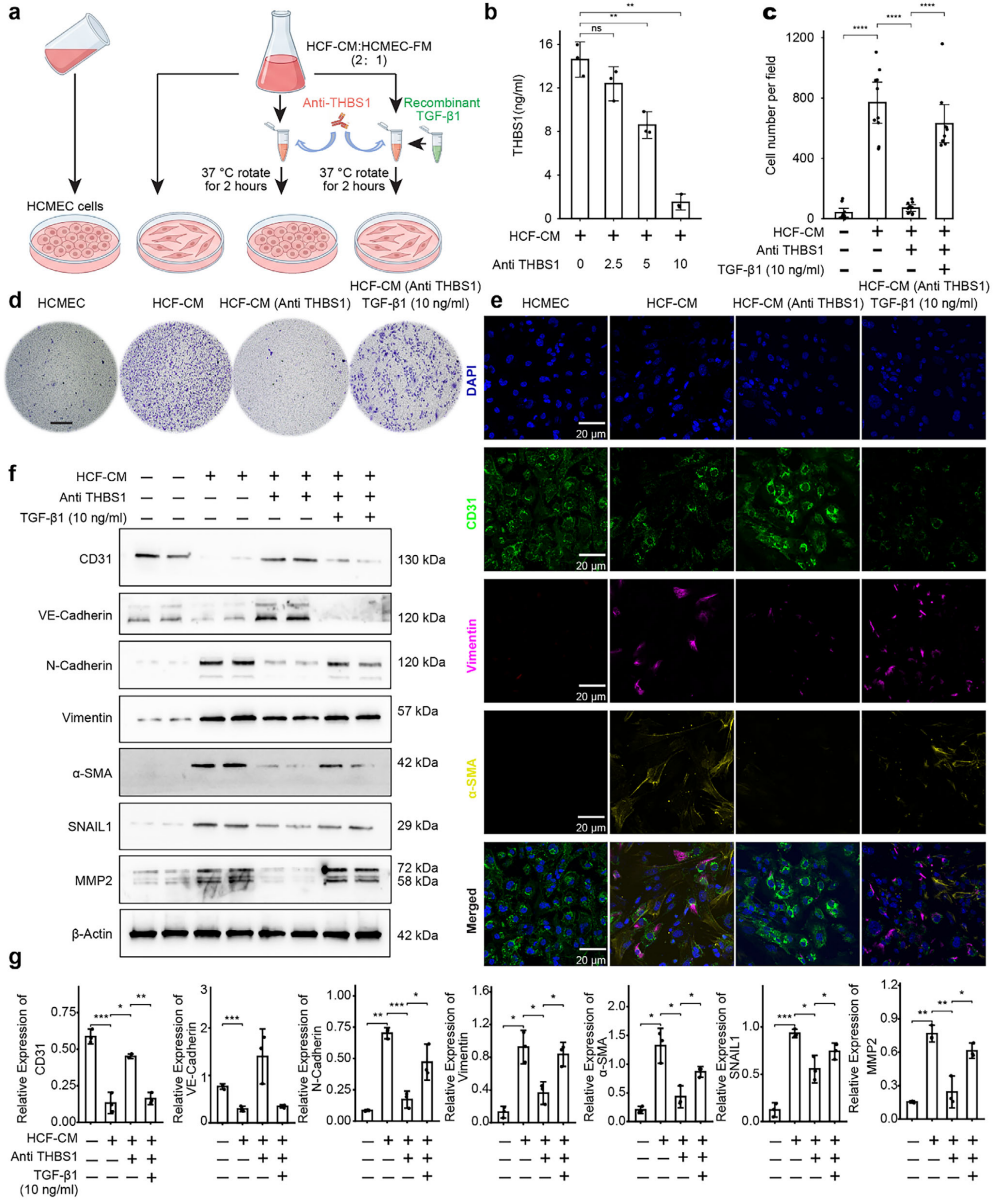
Functional inhibition and rescue confirm the indispensable role of the THBS1–TGF‐β1 axis in HCF‐induced EndoMT and migration. (a) Schematic of the experimental workflow for THBS1 functional perturbation and TGF‐β1 rescue assays in HCMECs. HCMECs were subjected to four conditions: baseline medium, HCF‐conditioned medium (HCF‐CM: HCMEC‐FM = 2:1), HCF‐CM pre‐incubated with neutralizing anti‐THBS1 antibody, or HCF‐CM co‐treated with anti‐THBS1 and recombinant TGF‐β1 at 37°C for 2 h prior to culture. (b) Validation of dose‐dependent THBS1 immunodepletion in HCF‐CM using neutralizing anti‐THBS1 antibody (2.5, 5, and 10 µg/mL), quantified by ELISA. Data are presented as mean ± 95% Cl (*n* = 3 biological replicates; ns, not significant, **p < 0.01 by two‐sided *Student's t‐test*). (c, d) Transwell cell migration assay showing that HCF‐CM‐stimulated HCMEC migration is markedly attenuated upon THBS1 neutralization and restored by exogenous TGF‐β1 supplementation (10 ng/mL). Representative crystal violet staining, Scale bars, 200 µm. (d) and statistical quantification of migrated cells per field (c) are shown. Data are presented as mean ± 95% Cl (*n* = 10 randomly selected fields; ****p < 0.0001 by two‐tailed unpaired *Student's t‐test*). (e) Representative immunofluorescence staining of HCMECs, demonstrating that THBS1 neutralization reverses the HCF‐CM‐induced downregulated CD31 (green) and upregulated Vimentin (magenta) / α‐SMA (yellow), whereas TGF‐β1 co‐treatment rescues the mesenchymal transition. Nuclei counterstained with DAPI (blue). Scale bars, 20 µm. (f, g) Western blot analysis (f) and quantitative protein profiling (g) of EndoMT markers (CD31, VE‐Cadherin, N‐Cadherin, Vimentin, α‐SMA, SNAIL1) and matrix metalloproteinase 2 (MMP2), confirming that THBS1 blockade blunts HCF‐CM‐driven EndoMT, which is rescued by TGF‐β1 pathway activation. β‐Actin served as loading control. Data are presented as mean ± 95% Cl (*n* = 3 independent experiments; *p < 0.05, **p < 0.01, and ***p < 0.001 by two‐sided *Student's t‐test*).

To evaluate whether the attenuated THBS1 signaling axis identified by scRNA‐seq analysis (Figure 5) mechanistically drives EndoMT beyond correlative predictions, we established an in vitro paracrine crosstalk assay using human cardiac fibroblasts (HCFs) and human cardiac microvascular endothelial cells (HCMECs) (Figure 6a and Figure S8a). We first harvested HCF‐conditioned medium (HCF‐CM) and treated HCMECs at increasing volumetric ratios of HCF‐CM to fresh endothelial complete medium (1:1, 2:1, and 3:1) for 48 h (Figure S8a). Bright‐field microscopy revealed a dose‐dependent morphological transition of HCMECs from a typical cobblestone endothelial monolayer to an elongated, spindle‐shaped mesenchymal phenotype (Figure S8b). While the 2:1 ratio effectively promoted mesenchymal transdifferentiation without compromising cell viability, the 3:1 ratio resulted in cellular detachment and altered morphology, likely attributable to nutrient insufficiency from excessive medium depletion (Figure S8b). Immunofluorescence (IF) confirmed that HCF‐CM treatment induced a progressive loss of the endothelial junction marker CD31 alongside a robust gain of the mesenchymal marker Vimentin (Figure S8c).

Establishing the 2:1 ratio as the optimal condition, we proceeded to dissect the specific role of THBS1 in this paracrine induction. We pre‐incubated HCF‐CM with a neutralizing anti‐THBS1 antibody (clone A4.1) [29], achieving near‐complete immunodepletion of soluble THBS1 at 10 ng/mL as validated by ELISA (p < 0.01; Figure 6a,b). In Transwell cell migration assays, HCF‐CM significantly augmented HCMEC migratory capacity compared to baseline medium (*p* < 0.0001; Figure 6c,d). Strikingly, specific immunodepletion of THBS1 markedly attenuated HCMEC migration, whereas co‐supplementation with recombinant TGF‐β1 (10 ng/mL) partially rescued the migratory phenotype under THBS1‐blocked conditions (*p* < 0.0001; Figure 6c,d). Confocal immunofluorescence imaging corroborated these findings: THBS1 blockade reversed the HCF‐CM‐induced downregulation of CD31 (green) and upregulation of mesenchymal filaments Vimentin (magenta) and α‐SMA (yellow), whereas TGF‐β1 co‐treatment partially restored the mesenchymal cell fate transition (Figure 6e).

Quantification of key molecular markers by Western blotting provided further protein‐level evidence (Figure 6f,g). HCF‐CM stimulation led to a marked suppression of endothelial identity markers (CD31 and VE‐cadherin) concomitant with an elevation of mesenchymal markers (N‐cadherin and Vimentin), key EndoMT factors (α‐SMA and SNAIL1), and the matrix metalloproteinase MMP2 (*p* < 0.05; Figure 6f,g). Importantly, THBS1 neutralization blunted these molecular shifts, preserving endothelial marker expression while suppressing mesenchymal and remodeling proteins (Figure 6f,g). Co‐treatment with exogenous TGF‐β1 following THBS1 blockade partially re‐induced the EndoMT cascade and restored MMP2 protein expression (Figure 6f,g). Taken together, these functional inhibition and rescue assays indicate that fibroblast‐derived THBS1 operates upstream of or in parallel with TGF‐β1 signaling to promote EndoMT and MMP2 execution, functionally driving the microenvironmental insufficiency associated with human VSD septal remodeling arrest.

### 2.7 Reference‐Based Spatial Projection Reveals Impaired Structural Maturation Signatures in the Muscular Ventricular Septum in VSD

**FIGURE 7:**
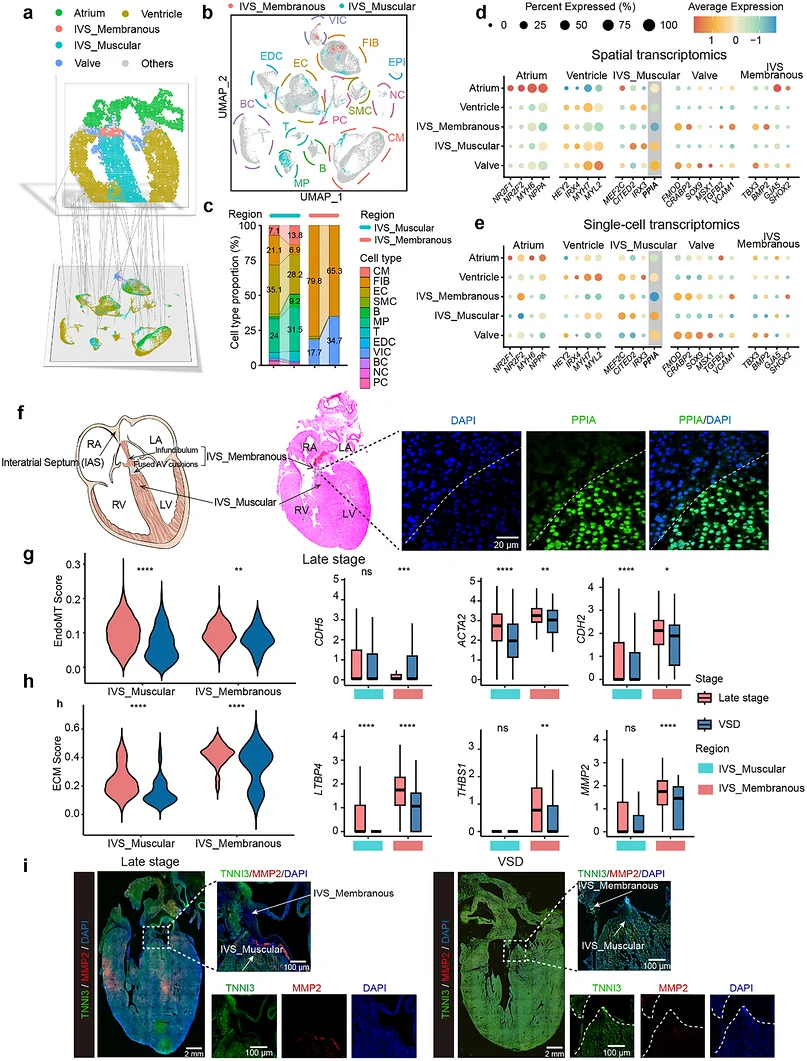
Reference‐based spatial projection reveals impaired structural maturation signatures in the muscular ventricular septum in VSD. (a, b) Integration of single‐cell and spatial transcriptomics. The schematic (a) illustrates the projection of single‐cell anchors onto spatial coordinates, generating a unified UMAP manifold (b) that resolves the anatomical distinction between the membranous (IVS_Membranous) and muscular (IVS_Muscular) ventricular septum. (c) Regional cellular composition quantified by bar charts, revealing an intrinsic enrichment of fibroblasts in the membranous septum compared to the cardiomyocyte‐dominated muscular region. (d, e) Identification of region‐specific markers visualized by dot plots of spatial (d) and single‐cell (e) expression profiles, defining the molecular signatures of membranous and muscular tissues. (f) Immunofluorescence validation of anatomical landmarks using PPIA (Cyclophilin A), confirming the spatial localization of identified markers in normal fetal hearts. Scale bars, 20 µm. (g) Global maturation defects across the septum, visualized by violin plots of EndoMT scores and box plots of key markers (e.g., CDH5, ACTA2). Analysis reveals a significant attenuation of these critical remodeling signatures in both membranous and muscular regions of VSD hearts compared to Late‐stage controls. ns, not significant, *p < 0.05, **p < 0.01, ****p < 0.0001 by two‐sided Wilcoxon tests. (h) Downregulation of key driver genes in the muscular ventricular septum in VSD. Box plots quantify the expression of ECM‐related factors (e.g., LTBP4, THBS1, MMP2). Notably, while LTBP4 is downregulated globally, MMP2 shows a specific and profound loss in the membranous region, linking region‐specific molecular deficits to the defect. ns, not significant, **p < 0.01, ****p < 0.0001 by two‐sided Wilcoxon tests. (i) In situ verification of the pathological mechanism by immunofluorescence demonstrated insufficient expression of MMP2 (red) in the muscular ventricular septum in VSD compared to the dense network observed in controls. The reduction was more pronounced in the developing membranous part of the IVS than in its muscular part. Scale bars, 100 µm.

Although scRNA‐seq elucidated cellular and molecular deficits in VSD, the spatial distribution of these perturbations remained to be defined. To address this, we constructed an integrated spatial molecular atlas by anchoring our single‐cell data onto a reference spatial transcriptomic map of the embryonic heart [30] (Figure 7a). This integration successfully deconvoluted the anatomical heterogeneity of the ventricular septum, resolving distinct membranous (IVS_Membranous) and muscular (IVS_Muscular) regions (Figure 7b,c).

To validate the fidelity of this spatial reconstruction, we interrogated the enrichment patterns of established regional markers. Dot plot visualization confirmed that the integrated clusters faithfully recapitulated canonical cardiac anatomy: contractile function genes (*MYH7*, *MYL2*) defined the ventricle‐associated clusters, whereas atrial identity factors (*NR2F1*, *MYH6*) were restricted to atrium‐associated regions. Furthermore, key regulators of valvular identity and ECM remodeling (*MSX1*, *TGFB2*, *FMOD*) were distinctly enriched in valve‐associated clusters. This precise regional segregation strongly corroborates the accuracy of our spatial assignment (Figure 7d,e). We also identified *PPIA* (Peptidylprolyl Isomerase A) as a novel spatially restricted marker. *PPIA* exhibited significantly higher expression in the muscular septum compared to the membranous region (Figure S9a–c). In situ immunofluorescence corroborated this spatial specificity at the protein level, establishing PPIA as a distinct molecular landmark for distinguishing septal sub‐regions during development (Figure 7f).

To infer the region‐specific alterations associated with septal failure, we projected the VSD‐associated molecular signatures onto the reference spatial atlas. Compared with the Late stage, the muscular septum in VSD predominantly exhibited inflammation‑driven stress responses, while the membranous septum showed more pronounced regional impairments in ECM remodeling (Figure S9d,e). Additionally, quantitative compositional analysis revealed a divergent remodeling pattern between the muscular and membranous components (Figure 7g,h). While fibroblasts were paradoxically enriched in the muscular septum of VSD hearts, they were significantly depleted in the membranous septum, the critical anatomical site of defect closure.

Spatially coincident with this inferred cellular depletion, the transcriptional signatures associated with the mesenchymal transition were profoundly suppressed. We observed a global attenuation of EndoMT activity scores across VSD septal regions (Figure 7g). Specifically, transcriptional profiling of the VSD membranous septum revealed an aberrant retention of endothelial identity markers (high *CDH5*/VE‐cadherin) concomitant with an attenuation in the induction of key mesenchymal drivers (*ACTA2*, *CDH2*/N‐cadherin), effectively mirroring the protein‐level block identified previously (Figure 5a).

Concomitantly, the ECM remodeling program exhibited a spatially restricted impairment (Figure 7h). Spatial projection inferred that the downregulation of critical regulators, including LTBP4, THBS1, and MMP2, was not uniform but was preferentially localized to the membranous septum. In situ immunofluorescence validation corroborated this topographic deficit, demonstrating a marked loss of MMP2 protein specifically within the VSD interventricular septum compared to the dense matrix network observed in controls (Figure 7i). Collectively, these computational spatial inferences, supported by orthogonal protein‐level validations, substantiate a model in which the localized attenuation of fibroblast‐endothelial crosstalk and ECM deposition within the developing membranous septum strongly precipitates structural remodeling arrest and septation anomalies.

## 3 Discussion and Conclusion

Congenital heart defects are a leading cause of neonatal morbidity, yet the molecular etiology of isolated perimembranous ventricular septal defects (VSD)—the most prevalent form—remains obscured by the scarcity of pathological specimens. The ventricular septum is a complex anatomical structure composed of distinct muscular and membranous components. The muscular septum forms through active proliferation and coalescence of cardiomyocytes from both ventricular heart fields [2, 3], followed by folding, apposition, and zipper‐like fusion of the medial compact ventricular walls during chamber ballooning [4]. Closure of the embryonic interventricular communication proceeds in multiple steps. The developing left ventricle first gains continuity with the aortic root via the secondary interventricular foramen. Subsequently, the precise alignment, fusion and myocardialisation of the proximal outflow cushions generate a muscular shelf that remodels into the free‐standing subpulmonary infundibular sleeve [5, 6]. A residual opening—the tertiary interventricular foramen—persists between the aortic root and right ventricle, and is definitively closed by the rightward margins of the atrioventricular cushions, the tubercles [5]. Subsequent delamination of the tricuspid septal leaflet partitions the nascent membranous septum into atrioventricular and interventricular components [31, 32]. Morphologically, a perimembranous ventricular septal defect arises specifically when developmental failure prevents the definitive closure of this tertiary interventricular foramen.

Current atlases predominantly map healthy trajectories [30, 33, 34, 35] or adult pathologies [15, 36, 37, 38], leaving a critical gap in our understanding of early human pathogenesis. Here, by constructing a single‐cell atlas of human isolated perimembranous VSD, we decode the disease not merely as an anatomical gap, but as a developmental disorder fundamentally driven by microenvironmental insufficiency and arrested structural remodeling. Our integrated analysis reveals a disturbed microenvironmental cascade driving septation failure. Initially, the stromal niche – defined as the non‑myocyte compartment containing endocardial cells, endothelial cells, fibroblasts, and their matrix – becomes depleted. This loss is accompanied by disrupted EndoMT and ECM remodeling, which in turn impose secondary metabolic and mechanical stress on the developing myocardium.

A central finding of our study is the identification of the non‐myocyte stromal niche as a primary driver in VSD pathogenesis. Contrary to the fibroblast expansion typical of adult heart failure [39, 40, 41], VSD hearts exhibit a profound depletion of fibroblasts and endothelial cells. This cellular imbalance suggests a failure in niche development rather than a reactive fibrotic response. Mechanistically, we trace this defect to a specific breakdown in endothelial‐fibroblast crosstalk. In the healthy developing heart, we identify a robust *THBS1*‐*CD36* signaling axis that instructs microvascular stability and remodeling. In VSD, this protective signal is abrogated and replaced by maladaptive *THBS1*‐*ITGA1* and *NPPA*‐*NPR1* stress signaling. This rewiring of the intercellular communication network provides a molecular explanation for the observed functional deficits: without the instructive cues from the fibroblast niche, endothelial cells fail to fully execute the EndoMT program required for septal fusion, as evidenced by the persistent expression of *CDH5* and the failure to induce mesenchymal drivers like *ACTA2*.

Our data further establish that this cellular communication failure is closely linked to a systemic collapse of the extracellular matrix (ECM) remodeling program. We observed a specific downregulation of the morphogenetic ECM module (Cluster 4), characterized by the loss of *THBS1*, *LTBP4*, and *MMP2*. The concurrent loss of TGF‐β1 activators (*THBS1*, *LTBP4*) and remodeling executors (*MMP2*) creates a “double hit” to the septal microenvironment: the tissue lacks both the signaling blueprint to guide fusion and the enzymatic machinery to execute physical remodeling. Furthermore, our in vitro functional perturbation and rescue assays using human cardiac primary cells confirmed that THBS1 blockade blunts HCF‐induced EndoMT and suppresses MMP2 expression, whereas TGF‐β1 supplementation partially restores this remodeling cascade. Reference‐based spatially resolved transcriptomics pinpoints this deficit preferentially to the membranous septum, the anatomical site most susceptible to defects. This localized molecular arrest—defined by the absence of *MMP2* and mesenchymal markers in the membranous region—provides a spatially precise molecular framework for membranous VSD formation in humans, which physically manifests as the failure to close the embryonic tertiary interventricular communication.

While our integrated transcriptomic data maps broad signaling and microenvironmental deficits across the perimembranous VSD, it is critical to acknowledge its inability to capture crucial spatial information of current methodologies in resolving distinct micro‐anatomical structures. To directly address this critical anatomical distinction, we explicitly differentiate the atrioventricular (AV) cushion tubercles from the proximal outflow cushions in terms of their developmental fates and contributions to septal morphogenesis. Specifically, the proximal outflow cushions are transient—they fuse and myocardialize between Carnegie stages 17 and 20, losing their septal identity and transforming into the muscular wall of the subpulmonary infundibular sleeve rather than a persistent partition [30, 42]. Moreover, we candidly discuss the technical limitation that our current single‑cell and spatial transcriptomic data cannot physically dissect the tubercles from the surrounding AV cushion mass: single‑cell dissociation destroys tissue architecture, while conventional spatial resolution remains insufficient. Capturing the brief developmental window when these cushions directly oppose—before outflow cushion myocardialization is complete—will require ultra‑high‑resolution spatial transcriptomics on early human embryos. This addition not only clarifies the anatomical precision of our interpretations but also appropriately acknowledges the current boundaries of our methodology.

Cardiomyocytes in hearts with deficient septation do not simply revert to an undifferentiated progenitor state. Instead, trajectory analysis reveals a bifurcation where ventricular cardiomyocytes are diverted from the canonical maturation path into a metabolically stressed, compensatory state (VCM_2). This state is characterized by the premature upregulation of load‐responsive genes (*NPPA*/*NPPB*) [43, 44] and oxidative phosphorylation pathways [24, 45], occurring asynchronously with structural maturation factors like *MEF2A*. Crucially, this phenotype differs fundamentally from adult heart failure: while adult failing hearts often show terminal dedifferentiation [46, 47], fetal ventricular cardiomyocytes under septal defect conditions exhibit a unique hypertrophic stress adaptation—a secondary compensatory attempt to maintain contractility in the face of structural defects and intracardiac shunting. This distinction highlights the plasticity of the fetal heart and suggests that early interventions targeting metabolic stress could potentially rescue myocardial function.

The clinical translational potential of our findings is supported by the preliminary identification of non‐invasive biomarkers. The strong correlation between reduced maternal serum levels of *MMP2*, *THBS1*, and *LTBP4* and fetal defect size in our expanded validation cohort suggests that these remodeling factors leak from the fetal circulation or reflect a systemic maternal‐fetal response to the defect. *MMP2*, in particular, emerges as a promising candidate for exploratory prenatal risk stratification, warranting further validation in larger multi‐center cohorts.

We acknowledge the limitations imposed by the unique nature of our study materials. The exceptional rarity and ethical complexities surrounding the acquisition of high‐fidelity human fetal VSD specimens inherently restrict our cohort size. While our single‐cell and spatial approach provide a high‐resolution snapshot of the pathological state, the causal hierarchies inferred here—particularly the specific dominance of the *THBS1* axis—warrant further functional validation in emerging human cardiac organoid models [48, 49, 50] or humanized animal systems, building upon our initial in vitro perturbation assays. Additionally, large‐scale prospective clinical cohorts and disease controls (e.g., other structural congenital heart defects) are needed to establish the sensitivity, specificity, and disease‐exclusive utility of maternal serum biomarkers across a broader spectrum of congenital heart defects.

Nevertheless, this work represents a valuable step forward in our understanding of human isolated perimembranous VSD. By framing the disease as a failure of stromal instruction and local EndoMT execution preventing the tubercles of the AV cushions from closing the tertiary interventricular foramen rather than primary myocardial dysfunction, we provide a spatially informed framework for cardiac dysmorphogenesis. Our data illuminate a critical “crosstalk‐remodeling axis”—anchored by THBS1‐mediated signaling, EndoMT progression, and MMP2‐driven matrix remodeling—whose collapse underpins the septal defect. This molecular insight not only clarifies the cellular origin of the anomaly but also highlights this axis as a compelling candidate for future therapeutic strategies aimed at promoting septal closure or mitigating secondary heart failure in neonates.

## 4 Materials and Methods

### 4.1 Ethics

This study was approved by the Ethics Committee of the First Hospital of Jilin University (Permit Nos. 2025–303, 2023‐HS‐107) and conducted in strict adherence to the Declaration of Helsinki and the International Society for Stem Cell Research (ISSCR) guidelines.

Prenatal fetal echocardiography was routinely performed prior to specimen collection. Clinical confirmation of isolated perimembranous VSD (*n* = 2 for scRNA‐seq and *n* = 2 for tissue validation; all at 22 post‐conceptional weeks, PCW) relied on a clear structural discontinuity between the membranous septum and the muscular crest beneath the septal tricuspid leaflet, accompanied by left‐to‐right shunting. We excluded fetuses presenting with complex proximal outflow cushions anomalies, major extracardiac defects, maternal systemic illnesses, or known teratogen exposures. Routine non‐invasive prenatal testing (NIPT) confirmed a low risk for common numerical trisomies (21, 18, and 13) in all VSD cases. Detailed clinical characteristics and donor metadata are compiled in Table S1.

### 4.2 Tissue Processing

Fresh cardiac tissues were dissected under sterile conditions within 2 h post‐procedure. For histological assessment, tissues were embedded in OCT compound, flash‐frozen in liquid nitrogen‐cooled isopentane, and sectioned at 10 µm.

### 4.3 Cell Culture and Conditioned Medium Preparation

Primary human cardiac fibroblasts (HCFs; Cat. ZQY100) and human cardiac microvascular endothelial cells (HCMECs; Cat. ZQY014) were obtained from Cell Research Laboratories (Shanghai, China). HCFs were cultured in complete Fibroblast Medium (Cat. ZMY100; ZQ‐XZ Bio), while HCMECs were maintained in complete Endothelial Cell Medium (Cat. ZMY014; ZQ‐XZ Bio). Cultures were kept at 37°C in a humidified 5% CO2 incubator, and cells from passages 3 through 6 were utilized across all functional experiments.

To generate HCF‐conditioned medium (HCF‐CM), primary HCFs were grown to 60% confluence in 75 cm^2^ flasks. After aspirating the culture medium and washing the cells twice with sterile PBS, the culture was refreshed with complete HCF medium. Following 72 h of incubation, the supernatant was collected, cleared of cellular debris by centrifugation (2000 rpm for 10 min at 4°C), filtered through a 0.22 µm PES membrane filter, and stored in aliquots at −80°C. For induction assays, HCF‐CM was mixed with fresh complete HCMEC medium at volume ratios of 1:1, 2:1, and 3:1. Optimization indicated that a 2:1 ratio effectively induced EndoMT without causing cytotoxicity or cell detachment; thus, this proportion was adopted for all subsequent assays.

### 4.4 In Vitro THBS1 Functional Neutralization and TGF‐β1 Rescue Assays

To block THBS1 function, HCF‐CM was pre‐incubated with a neutralizing mouse monoclonal anti‐THBS1 antibody (clone A4.1; Thermo Fisher Scientific, MA5‐13377) at increasing concentrations (2.5, 5, and 10 ng/mL) for 2 h at 37°C with gentle rotation prior to addition to HCMEC cultures. THBS1 depletion in the conditioned medium was confirmed using a Human THBS1 ELISA kit (COIBO, CB11586‐Hu), and prior to the assay, samples were concentrated to 10‐fold of the original volume using ultrafiltration tubes.

For rescue experiments, HCMECs were assigned to four treatment conditions: (1) Baseline control (fresh complete HCMEC medium); (2) HCF‐CM (HCF‐CM: HCMEC medium = 2:1); (3) THBS1‐blocked (HCF‐CM pre‐incubated with 10 ng/mL anti‐THBS1 antibody); and (4) TGF‐β1 rescue (HCF‐CM with 10 ng/mL anti‐THBS1 antibody plus 10 ng/mL recombinant human TGF‐β1; R&D Systems, 240‐B). Cells were harvested after 48 h for migration, immunofluorescence, and Western blot analyses.

### 4.5 H&E and WGA Staining

Sections were stained with hematoxylin and eosin (H&E) for morphological verification or with Alexa Fluor 488‑conjugated Wheat Germ Agglutinin (G1730, Servicebio) to visualize cardiomyocyte boundaries.

### 4.6 Immunofluorescence Staining

Sections were fixed in 4% paraformaldehyde for 30 min at room temperature, permeabilized with 0.5% Triton X‑100 and blocked in 5% normal donkey serum. Primary antibodies targeting MMP2 (ab92536, Abcam), Cyclophilin A (2175S, CST), Vimentin (10366‐1‐AP; Proteintech), α‐SMA (14395‐1‐AP; Proteintech), CD31 (55274, BD biosciences), CD20 (ab78237; Abcam), NKX2‐5 (13921‐1‐AP, Proteintech), TNNI3 (ab56357, Abcam) N‑cadherin (22018‑1‑AP, Proteintech), and VE‑cadherin (83766‑6‑RR, Proteintech) were incubated at 4°C, followed by fluorophore‐conjugated secondary antibodies. Nuclei were counterstained with DAPI. Images were acquired using a Nikon AXR (Ti2‐E) confocal microscope.

### 4.7 Transwell Cell Migration Assay

HCMEC migration was assessed using 24‐well Transwell inserts (8.0 µm pore size; Corning, 3422). Following 48 h of treatment, cells were trypsinized, washed, and resuspended in serum‐free medium. We seeded 5 × 10^4^ cells in 200 µL of serum‐free medium into the upper chamber, while filling the lower chamber with 600 µL of the corresponding treatment media (Baseline, HCF‐CM, HCF‐CM + anti‐THBS1, or HCF‐CM + anti‐THBS1 + recombinant TGF‐β1).

After 48 h of incubation at 37°C, non‐migrating cells on the upper surface were gently removed using a cotton swab. Cells that migrated to the lower surface were fixed with 4% paraformaldehyde for 20 min and stained with 0.1% crystal violet for 15 min. Images were captured on an inverted microscope (Olympus IX73), and cell migration was quantified by averaging counts from 3 to 4 random microscopic fields per insert across 3 independent experiments.

### 4.8 ELISA Quantification of Serum Biomarkers

Maternal peripheral blood samples were collected from residual clinical specimens under ethical approval. Serum was isolated by centrifugation (2000 × *g*, 15 min, 4°C) and stored at −80°C. Concentrations of THBS1, MMP2, and LTBP4 were quantified using human‐specific ELISA kits (COIBO; THBS1: cat. CB11586‑Hu, MMP2: cat. CB10745‑Hu, LTBP4: cat. CB20253‑Hu) following the manufacturer's protocols. All samples were performed in duplicate, and absolute concentrations were calculated against standard curves.

### 4.9 Western Blotting

Cells from the four treatment groups were lysed in RIPA buffer (Beyotime) supplemented with 1× protease and phosphatase inhibitors (Roche) for 30 min on ice. Lysates were clarified by centrifugation at 14 000 × *g* for 15 min at 4°C, and protein concentrations were determined using a BCA assay (Thermo Fisher Scientific). Protein samples (20 µg/lane) were boiled in 5× SDS loading buffer for 10 min, separated by 10% or 12% SDS‐PAGE, and transferred onto 0.22 µm PVDF membranes (Millipore).

Membranes were blocked with 5% non‐fat milk in TBST for 1 h at room temperature and incubated overnight at 4°C with primary antibodies against CD31 (1:1000; Abcam), VE‐cadherin (1:1000; Proteintech), N‐cadherin (1:1000; Proteintech), Vimentin (1:1000; Proteintech), α‐SMA (1:1000; Proteintech), SNAIL1 (1:800; CST), MMP2 (1:1000; Abcam), and β‐Actin (1:5000; Proteintech) as the loading control. After TBST washes, membranes were incubated with HRP‐conjugated secondary antibodies (1:5000; Proteintech) for 1 h at room temperature. Signals were developed using ECL substrate (Bio‐Rad) and visualized on a ChemiDoc MP Imaging System (Bio‐Rad). Densitometry was performed with Image Lab software (v6.1, Bio‐Rad) from *n* = 3 independent biological replicates. Uncropped blot images are available in the Supporting Information.

### 4.10 Single‐Cell Suspension Preparation

Fresh heart tissues were minced and dissociated using a refined enzymatic protocol optimized for early developmental stages. Tissues were digested in Earle's Balanced Salt Solution (EBSS; Worthington Biochemical, LK003188) containing Collagenase II (200 U mL^−^^1^; Worthington Biochemical, LS004174) and DNase I (1 mg mL^−^^1^; Worthington Biochemical, LK003170) at 37°C.

Digestion duration ranged from 45 min to 2.5 h, depending on tissue density. Mechanical dissociation was performed at approximately 25‐min intervals using fire‐polished Pasteur pipettes with progressively decreasing bore sizes. Following digestion, the cell suspension was filtered through a 30 µm cell strainer (CellTrics, Sysmex) and quenched with EBSS. To minimize ambient RNA contamination and remove erythrocytes, a gradient centrifugation step using an albumin inhibitor solution (Ovomucoid protease inhibitor with BSA, OI‐BSA, LK003182) was performed when necessary. Cell concentration and viability were verified prior to loading onto the MGI C4 platform (MGI Tech) for scRNA‐seq library construction.

### 4.11 scRNA‐seq Data Processing, Integration, and Clustering

Raw sequencing reads were aligned to the GRCh37 reference genome using DNBC4tools (v2.1.3). Downstream analyses including filtering, normalization, dimensionality reduction, and clustering were performed using Seurat (v4.4.0). Rigorous quality control (QC) criteria were applied to remove low‐quality cells and potential doublets. Cells expressing fewer than 200 genes or those expressed in fewer than 3 cells were discarded. Sample‐specific filtering was applied to address technical heterogeneity: for dataset VSD_S2, cells with extreme gene counts (<1000 or >5000) or the mitochondrial content >5% were excluded.

After QC, data were scaled, and Principal Component Analysis (PCA) was performed using the top 2000 highly variable genes. The first 30 principal components (PCs) were subsequently utilized to drive Uniform Manifold Approximation and Projection (UMAP) dimensionality reduction and unsupervised graph‐based clustering (resolution = 0.7). Doublet detection was conducted using Scrublet (v0.2.3) with an expected doublet rate of 0.06, and identified doublets were removed. Cluster identity was assigned by manual annotation of cluster‐specific marker genes (FindAllMarkers).

For high‐resolution subpopulation analysis, Endothelial Cells (EC), Fibroblasts (FIB), Cardiomyocytes (CM), Smooth Muscle Cells (SMC), and B cells were re‐clustered with optimized resolutions: CM (0.7), EC (0.1), FIB (0.2), B (0.2), and SMC (0.2).

### 4.12 Differential Abundance Analysis

To identify significant shifts in cell type composition between groups, differential abundance (DA) analysis was performed using the miloR package (v2.5.1). Neighborhoods were constructed with a sampling proportion (prop) of 0.2, a k‐nearest neighbor (k) parameter of 30, and 30 reduced dimensions (d). Statistical significance was assessed using False Discovery Rate (FDR) control. Neighborhoods exhibiting an FDR < 0.05 and the log2 fold change (log2FC) > 0.1 were defined as significantly differentially abundant.

### 4.13 Differential Expression and CHD Risk Gene Enrichment

Differentially expressed genes (DEGs) were identified using the Wilcoxon rank‐sum test implemented in Seurat. Inclusion criteria were set to a minimum log‐fold change of 0.1, a Bonferroni‐corrected *p*‐value < 0.05, and a minimum detection percentage of 10% (min.pct = 0.1). Biological functions of DEGs were characterized via Gene Ontology (GO) enrichment analysis using clusterProfiler (v4.10.1), with multiple testing controlled via the False Discovery Rate (FDR) method (adjusted *P* < 0.05).To link cell subpopulations to clinical phenotypes, we curated a comprehensive panel of Congenital Heart Disease (CHD) associated candidate genes spanning six major categories: Atrioventricular Septal Defects (*n* = 37), Atrial Septal Defects (*n* = 141), Malformation of the Outflow Tract (*n* = 116), Valve Defects (*n* = 137), Single Ventricle Disease (*n* = 24), and Ventricular Septal Defects (*n* = 141) [12, 13, 14, 15] Enrichment of these phenotype‐specific gene sets within the marker genes of each cell subpopulation was evaluated using a one‐sided Fisher's exact test, with significance defined as *P* < 0.05.

### 4.14 Trajectory Inference and Cross‐Stage Alignment

Developmental trajectories were reconstructed using Monocle3 (v1.4.26). Louvain clusters derived from Scanpy were used to initialize the trajectory graph. Pseudotime trajectories were calculated to represent continuous cellular transitions spanning early, mid, and late developmental stages, as well as VSD samples. To systematically compare developmental progression between healthy and pathological states, trajectory alignment was performed using the Genes2Genes dynamic programming framework.

### 4.15 Temporal Dynamics of ECM Gene Expression

ECM dynamics were interrogated using a candidate set of 1027 ECM‐related genes from MatrisomeDB 2.0 [51], filtering for 164 ECM genes expressed in at least one developmental stage. A pseudo‐bulk matrix was generated by calculating the average expression of these genes across the four study groups (Early, Mid, Late, and VSD). Temporal expression patterns were resolved by performing soft clustering on the normalized average expression data using the fuzzy c‐means algorithm from the Mfuzz package (v2.66.0), with the cluster number set to 4.

### 4.16 Intercellular Communication Networks Analysis

Cell‐cell signaling interactions were inferred using CellChat (v2.2.0). By leveraging the CellChatDB human ligand‐receptor database, communication probabilities were estimated among annotated cell populations. These probabilities were calculated using a mass action model based on the truncated mean of gene expression. To ensure robust inference, cell clusters comprising fewer than 10 cells were excluded. The statistical significance of the inferred communication networks was determined by empirical p‐values (*P* < 0.05), derived from a permutation test utilizing 100 random permutations. To identify deregulated signaling axes, datasets from Late stage controls and VSD samples were integrated for comparative analysis. Differential communication networks were identified based on a minimum expression threshold of 10% and a fold‐change exceeding 0.1. Statistical significance was defined using a Bonferroni‐corrected *P*‐value < 0.05.

### 4.17 Single‐Cell Gene Signature and Pathway Scoring

To quantify specific biological activities at single‐cell resolution, gene set activity analysis was performed using the AUCell package (v1.25.2). Two specific gene signatures were defined: (1) an ECM signature, derived from genes classified into Cluster 3 of the temporal clustering analysis; and (2) an EndoMT signature, comprising 49 canonical markers curated from published literature [26]. Activity scores for these signatures were calculated for each cell and statistical differences between groups were assessed using the Wilcoxon rank‐sum test.

### 4.18 Spatial Mapping via Multimodal Integration

Single‐cell transcriptomes were spatially contextualized through a computational projection strategy by integrating with a previously published healthy human fetal heart reference Spatial Transcriptomics (ST) dataset at 12 PCW [30] using Seurat. The ST reference was segmented into anatomical regions: Atrium, Ventricle, IVS_Membranous, IVS_Muscular, Valve, and Others. Integration anchors between the scRNA‐seq (query) and ST (reference) datasets were identified using the FindTransferAnchors function with Canonical Correlation Analysis (CCA) reduction (dims = 1–30). Spatial labels were projected onto single cells using the TransferData function based on PCA‐weighted anchors. To refine localization accuracy, cells initially classified as “Others” were reassigned to their second‑highest scoring anatomical region if the prediction score exceeded a confidence threshold of 0.2.

### 4.19 Functional Enrichment of Septal Subpopulations

Functional disparities within the IVS_Muscular and IVS_Membranous regions between Late stage and VSD groups were analyzed via Gene Set Enrichment Analysis (GSEA). A pre‐ranked gene list was generated based on the average log2 fold change of all detected genes (FindMarkers). GSEA was executed using the clusterProfiler package against the KEGG Medicus (v2025.1) gene sets from the MsigDB database (https://www.gsea‑msigdb.org/gsea/msigdb). Significant pathways were defined by an absolute Normalized Enrichment Score (NES) > 1 and an adjusted p‑value < 0.05.

### 4.20 Statistical Analysis

Statistical analyses were conducted using R software (version 4.4.3). Continuous variables between two groups were compared using the two‐tailed *Student's t‐test* or the *Wilcoxon rank‐sum test*, with statistical significance defined as *P* < 0.05. Associations between variables were evaluated using Pearson or Spearman correlation analyses, as appropriate. No statistical methods were used to pre‑determine sample size for any of the analyses in this study.

## Author Contributions

Y.Y. conceived and designed the study. X.Z. designed and performed experiments supervised by Y.Y. and X.Y.; L.Z. and X.Z. performed computational analyses supervised by Y.Y and H.X.; Z.S. provided technical support. X.Z., L.Z. and Y.Y. wrote the paper. All authors participated in data discussion and interpretation.

## Funding

We thank all members of the Yue lab for critical comments on this manuscript. We thank the Imaging Platform of the Public Experimental Platform, at First Hospital of Jilin University for technical help. Y.Y. was supported by grants from the National Key R&D Program of China (2022YFA1106200) and the National Natural Science Foundation of China (32370873 and 32522028), the Natural Science Foundation of Jilin Province, China (20250101028JJ), the Bethune Medical Department of Jilin University (4044450001 and 4047330002), and the Fundamental Research Funds for the Central Universities, JLU (419021425Y11). X.Y was supported by Jilin Provincial Department of Science and Technology (YDZJ202501ZYTS726). H.X. was supported by the Non‐profit Central Research Institute Fund of Chinese Academy of Medical Sciences (2022‐RC180‐07); CAMS Innovation Fund for Medical Sciences (CIFMS) (2025‐I2M‐XHXX‐138, 2023‐I2M‐2‐007, 2022‐I2M‐1‐022); The National Natural Science Foundation of China (32470884).

## Conflicts of Interest

The authors declare no conflicts of interest.

## Supporting information




**Supporting File 1**: advs78016‐sup‐0001‐SuppMat.pdf.

**Supporting File 2**: advs78016‐sup‐0002‐SuppMat.xlsx.

## Data Availability

The single‐cell RNA sequencing data generated in this study are deposited in the Genome Sequence Archive for Humans (GSA‐Human: HRA015439) and are publicly accessible at https://ngdc.cncb.ac.cn/gsa‐human/browse/HRA015439. Publicly available datasets used in this study were obtained from the archived resources provided in their original publications under the following links https://data.mendeley.com/datasets/fhtb99mdzd/1. Source data are provided in this paper.

## References

[1] Y. Li , J. Du , S. Deng , et al., “The Molecular Mechanisms of Cardiac Development and Related Diseases,” Signal Transduction and Targeted Therapy 9, no. 1 (2024): 368, 10.1038/s41392-024-02069-8. PMC11666744 39715759

[2] S. Zaffran , R. G. Kelly , S. M. Meilhac , M. E. Buckingham , and N. A. Brown , “Right Ventricular Myocardium Derives From the Anterior Heart Field,” Circulation Research 95, no. 3 (2004): 261–268, 10.1161/01.Res.0000136815.73623.Be. 15217909

[3] D. Franco , S. M. Meilhac , V. M. Christoffels , A. Kispert , M. Buckingham , and R. G. Kelly , “Left and Right Ventricular Contributions to the Formation of the Interventricular Septum in the Mouse Heart,” Developmental Biology 294, no. 2 (2006): 366–375, 10.1016/j.ydbio.2006.02.045. 16677630

[4] T. H. Bønnelykke , C. Coulon , R. Sturny , et al., “Morphogenesis of the Muscular Ventricular Septum of the Mouse Heart Is Driven by Retinoic Acid Signalling to Myocardium,” BioRxiv (2026), 10.64898/2026.08.12.744354.

[5] R. H. Anderson , J. Kerwin , W. H. Lamers , et al., “Cardiac Development Demystified by Use of the HDBR Atlas,” Journal of Anatomy 245, no. 4 (2024): 517–534, 10.1111/joa.14066. PMC11424819 38783643

[6] J. Hikspoors , N. Kruepunga , G. M. C. Mommen , S. E. Köhler , R. H. Anderson , and W. H. Lamers , “A Pictorial Account of the Human Embryonic Heart Between 3.5 and 8 Weeks of Development,” Communications Biology 5, no. 1 (2022): 226, 10.1038/s42003-022-03153-x. PMC8917235 35277594

[7] B. Hogers , M. C. DeRuiter , A. C. Gittenberger‐de Groot , and R. E. Poelmann , “Unilateral Vitelline Vein Ligation Alters Intracardiac Blood Flow Patterns and Morphogenesis in the Chick Embryo,” Circulation Research 80, no. 4 (1997): 473–481, 10.1161/01.res.80.4.473. 9118477

[8] V. Garg , I. S. Kathiriya , R. Barnes , et al., “GATA4 Mutations Cause Human Congenital Heart Defects and Reveal an Interaction With TBX5,” Nature 424, no. 6947 (2003): 443–447, 10.1038/nature01827. 12845333

[9] J. He , Y. Yang , R. Jiang , et al., “Integration of Single‐Cell and Spatial Transcriptomics by SEU‐TCA Reveals the Spatial Origin of Early Cardiac Progenitors,” Genome Biology 26, no. 1 (2025): 158, 10.1186/s13059-025-03633-3. PMC12150484 40495257

[10] T. A. R. Ramos , S. Urquiza‐Zurich , S. Y. Kim , et al., “Single‐Cell Transcriptional Landscape of Long Non‐Coding RNAs Orchestrating Mouse Heart Development,” Cell Death & Disease 14, no. 12 (2023): 841, 10.1038/s41419-023-06296-9. PMC10728149 38110334

[11] Q. Wang , T. M. Tang , M. Youlton , et al., “Epistasis Regulates Genetic Control of Cardiac Hypertrophy,” Nature Cardiovascular Research 4, no. 6 (2025): 740–760, 10.1038/s44161-025-00656-8. PMC12170338 40473955

[12] X. R. Ma , S. D. Conley , M. Kosicki , et al., “Molecular Convergence of Risk Variants for Congenital Heart Defects Leveraging a Regulatory Map of the Human Fetal Heart,” medRxiv (2024), 10.1101/2024.11.20.24317557.

[13] S. U. Morton , D. Quiat , J. G. Seidman , and C. E. Seidman , “Genomic Frontiers in Congenital Heart Disease,” Nature Reviews Cardiology 19, no. 1 (2022): 26–42, 10.1038/s41569-021-00587-4. PMC9236191 34272501

[14] S. C. Jin , J. Homsy , S. Zaidi , et al., “Contribution of Rare Inherited and De Novo Variants in 2,871 Congenital Heart Disease Probands,” Nature Genetics 49, no. 11 (2017): 1593–1601, 10.1038/ng.3970. PMC5675000 28991257

[15] A. L. Koenig , I. Shchukina , J. Amrute , et al., “Single‐Cell Transcriptomics Reveals Cell‐Type‐Specific Diversification in Human Heart Failure,” Nature Cardiovascular Research 1, no. 3 (2022): 263–280, 10.1038/s44161-022-00028-6. PMC9364913 35959412

[16] R. O. Hynes and A. Naba , “Overview of the Matrisome–an Inventory of Extracellular Matrix Constituents and Functions,” Cold Spring Harbor Perspectives in Biology 4, no. 1 (2012): a004903, 10.1101/cshperspect.a004903. PMC3249625 21937732

[17] D. G. M. Molin , U. Bartram , K. Van der Heiden , et al., “Expression Patterns of Tgfβ1–3 Associate With Myocardialisation of the Outflow Tract and the Development of the Epicardium and the Fibrous Heart Skeleton,” Developmental Dynamics 227, no. 3 (2003): 431–444, 10.1002/dvdy.10314. 12815630

[18] L. A. Compton , D. A. Potash , N. A. Mundell , and J. V. Barnett , “Transforming Growth Factor‐β Induces Loss of Epithelial Character and Smooth Muscle Cell Differentiation in Epicardial Cells,” Developmental Dynamics 235, no. 1 (2006): 82–93, 10.1002/dvdy.20629. 16258965

[19] S. L. Dallas , K. Miyazono , T. M. Skerry , G. R. Mundy , and L. F. Bonewald , “Dual Role for the Latent Transforming Growth Factor‐beta Binding Protein in Storage of Latent TGF‐Beta in the Extracellular Matrix and as a Structural Matrix Protein,” The Journal of Cell Biology 131, no. 2 (1995): 539–549, 10.1083/jcb.131.2.539. PMC2199978 7593177

[20] J. Sun , X. Ge , Y. Wang , L. Niu , L. Tang , and S. Pan , “USF2 Knockdown Downregulates THBS1 to Inhibit the TGF‐β Signaling Pathway and Reduce Pyroptosis in Sepsis‐Induced Acute Kidney Injury,” Pharmacological Research 176 (2022): 105962, 10.1016/j.phrs.2021.105962. 34756923

[21] D. Vanhoutte , T. G. Schips , R. A. Minerath , et al., “Thbs1 Regulates Skeletal Muscle Mass in a TGFβ‐Smad2/3‐ATF4‐Dependent Manner,” Cell Reports 43, no. 5 (2024): 114149, 10.1016/j.celrep.2024.114149. PMC11217783 38678560

[22] H. Ayabe , E. A. K. DePasquale , S. P. Amarachintha , et al., “Cellular Crosstalk Mediated by TGF‐β Drives Epithelial‐Mesenchymal Transition in Patient‐Derived Multi‐Compartment Biliary Organoids,” Nature Communications 16, no. 1 (2025): 6575, 10.1038/s41467-025-61442-5. PMC12271497 40675983

[23] J. Ma , G. Sanchez‐Duffhues , M. J. Goumans , and P. T. Dijke , “TGF‐β‐Induced Endothelial to Mesenchymal Transition in Disease and Tissue Engineering,” Frontiers in Cell and Developmental Biology 8 (2020): 260, 10.3389/fcell.2020.00260. PMC7187792 32373613

[24] F. Peng , M. Liao , W. Jin , et al., “2‐APQC, a Small‐Molecule Activator of Sirtuin‐3 (SIRT3), Alleviates Myocardial Hypertrophy and Fibrosis by Regulating Mitochondrial Homeostasis,” Signal Transduction and Targeted Therapy 9, no. 1 (2024): 133, 10.1038/s41392-024-01816-1. PMC11094072 38744811

[25] D. Medici and R. Kalluri , “Endothelial–Mesenchymal Transition and its Contribution to the Emergence of Stem Cell Phenotype,” Seminars in Cancer Biology 22, no. 5‐6 (2012): 379–384, 10.1016/j.semcancer.2012.04.004. PMC3422405 22554794

[26] Y. Yoshimatsu and T. Watabe , “Emerging Roles of Inflammation‐Mediated Endothelial–Mesenchymal Transition in Health and Disease,” Inflammation and Regeneration 42, no. 1 (2022): 9, 10.1186/s41232-021-00186-3. PMC8818500 35130955

[27] P. Dong , M. Kaneuchi , H. Watari , S. Sudo , and N. Sakuragi , “MicroRNA‐106b Modulates Epithelial–Mesenchymal Transition by Targeting TWIST1 in Invasive Endometrial Cancer Cell Lines,” Molecular Carcinogenesis 53, no. 5 (2014): 349–359, 10.1002/mc.21983. 24002805

[28] S. S. Ladak , L. W. McQueen , K. Tomkova , G. R. Layton , G. J. Murphy , and M. Zakkar , “Acute Shear Stress Induces TWIST‐Mediated EndMT in Venous Endothelial Cells and Human Long Saphenous Veins,” Cells 14, no. 17 (2025): 1369, 10.3390/cells14171369. PMC12428368 40940783

[29] G. Jacquemin , A. Wurmser , M. Huyghe , et al., “Paracrine Signalling Between Intestinal Epithelial and Tumour Cells Induces a Regenerative Programme,” Elife 11 (2022): e76541, 10.7554/eLife.76541. PMC9094746 35543624

[30] E. Lázár , R. Mauron , Z. Andrusivová , et al., “Spatiotemporal Gene Expression and Cellular Dynamics of the Developing Human Heart,” Nature Genetics 57, no. 11 (2025): 2756–2771, 10.1038/s41588-025-02352-6. PMC12597827 41162788

[31] L. H. Van Mierop , R. D. Alley , H. W. Kausel , and A. Stranahan , “The Anatomy and Embryology of Endocardial Cushion Defects,” The Journal of Thoracic and Cardiovascular Surgery 43 (1962): 71–83. 13924605

[32] A. C. Wenink and A. C. Gittenberger‐de Groot , “The Role of Atrioventricular Endocardial Cushions in the Septation of the Heart,” International Journal of Cardiology 8, no. 1 (1985): 25–44, 10.1016/0167-5273(85)90261-x. 3997290

[33] M. Asp , S. Giacomello , L. Larsson , et al., “A Spatiotemporal Organ‐Wide Gene Expression and Cell Atlas of the Developing Human Heart,” Cell 179, no. 7 (2019): 1647–1660, 10.1016/j.cell.2019.11.025. 31835037

[34] E. N. Farah , R. K. Hu , C. Kern , et al., “Spatially Organized Cellular Communities Form the Developing Human Heart,” Nature 627, no. 8005 (2024): 854–864, 10.1038/s41586-024-07171-z. PMC10972757 38480880

[35] M. Sahara , F. Santoro , J. Sohlmér , et al., “Population and Single‐Cell Analysis of Human Cardiogenesis Reveals Unique LGR5 Ventricular Progenitors in Embryonic Outflow Tract,” Developmental Cell 48, no. 4 (2019): 475–490, 10.1016/j.devcel.2019.01.005. 30713072

[36] M. Chaffin , I. Papangeli , B. Simonson , et al., “Single‐Nucleus Profiling of Human Dilated and Hypertrophic Cardiomyopathy,” Nature 608, no. 7921 (2022): 174–180, 10.1038/s41586-022-04817-8. PMC12591363 35732739

[37] T. Pan , L. Lu , K. Youker , et al., “Single‐Cell Splicing Isoform Atlas of the Adult Human Heart and Heart Failure,” Circulation 152, no. 21 (2025): 1501–1514, 10.1161/CIRCULATIONAHA.125.074959. PMC12574690 41017471

[38] L. Wang , P. Yu , B. Zhou , et al., “Single‐Cell Reconstruction of the Adult Human Heart During Heart Failure and Recovery Reveals the Cellular Landscape Underlying Cardiac Function,” Nature Cell Biology 22, no. 1 (2020): 108–119, 10.1038/s41556-019-0446-7. 31915373

[39] M. Fu , S. Jia , L. Xu , et al., “Single‐Cell Multiomic Analysis Identifies Macrophage Subpopulations in Promoting Cardiac Repair,” Journal of Clinical Investigation 134, no. 19 (2024): e175297, 10.1172/JCI175297. PMC11444165 39190625

[40] R. Patrick , V. Janbandhu , V. Tallapragada , et al., “Integration Mapping of Cardiac Fibroblast Single‐Cell Transcriptomes Elucidates Cellular Principles of Fibrosis in Diverse Pathologies,” Science Advances 10, no. 25 (2024): adk8501, 10.1126/sciadv.adk8501. PMC11192082 38905342

[41] L. Weng , J. Ye , F. Yang , et al., “TGF‐β1/SMAD3 Regulates Programmed Cell Death 5 That Suppresses Cardiac Fibrosis Post–Myocardial Infarction by Inhibiting HDAC3,” Circulation Research 133, no. 3 (2023): 237–251, 10.1161/CIRCRESAHA.123.322596. 37345556

[42] R. H. Anderson , W. H. Lamers , J. P. J. M. Hikspoors , et al., “Development of the Arterial Roots and Ventricular Outflow Tracts,” Journal of Anatomy 244, no. 3 (2024): 497–513, 10.1111/joa.13973. PMC10862166 37957890

[43] J. R. Becker , T. Y. Robinson , C. Sachidanandan , et al., “In Vivo Natriuretic Peptide Reporter Assay Identifies Chemical Modifiers of Hypertrophic Cardiomyopathy Signalling,” Cardiovascular Research 93, no. 3 (2012): 463–470, 10.1093/cvr/cvr350. PMC3410427 22198505

[44] J. Gao , M. Liu , M. Lu , et al., “Integrative Analysis of Transcriptome, DNA Methylome, and Chromatin Accessibility Reveals Candidate Therapeutic Targets in Hypertrophic Cardiomyopathy,” Protein & Cell 15, no. 11 (2024): 796–817, 10.1093/procel/pwae032. PMC11528543 38780967

[45] S. Ranjbarvaziri , K. B. Kooiker , M. Ellenberger , et al., “Altered Cardiac Energetics and Mitochondrial Dysfunction in Hypertrophic Cardiomyopathy,” Circulation 144, no. 21 (2021): 1714–1731, 10.1161/CIRCULATIONAHA.121.053575. PMC8608736 34672721

[46] Y.‐Y. Cheng , Z. Gregorich , R. P. Prajnamitra , et al., “Metabolic Changes Associated With Cardiomyocyte Dedifferentiation Enable Adult Mammalian Cardiac Regeneration,” Circulation 146, no. 25 (2022): 1950–1967, 10.1161/CIRCULATIONAHA.122.061960. PMC9808601 36420731

[47] X. Li , F. Wu , S. Günther , et al., “Inhibition of Fatty Acid Oxidation Enables Heart Regeneration in Adult Mice,” Nature 622, no. 7983 (2023): 619–626, 10.1038/s41586-023-06585-5. PMC10584682 37758950

[48] P. Hofbauer , S. M. Jahnel , N. Papai , et al., “Cardioids Reveal Self‐Organizing Principles of Human Cardiogenesis,” Cell 184, no. 12 (2021): 3299–3317, 10.1016/j.cell.2021.04.034. 34019794

[49] Y. R. Lewis‐Israeli , A. H. Wasserman , M. A. Gabalski , et al., “Self‐Assembling Human Heart Organoids for the Modeling of Cardiac Development and Congenital Heart Disease,” Nature Communications 12, no. 1 (2021): 5142, 10.1038/s41467-021-25329-5. PMC8390749 34446706

[50] H. Y. Zhao , J. B. Jiang , S. N. Wang , and C. Y. Miao , “Technologies and Applications of Current Human Cardiac Organoids,” Acta Pharmaceutica Sinica B 15, no. 11 (2025): 5734–5757, 10.1016/j.apsb.2025.09.013. PMC12648008 41311381

[51] X. Shao , C. D. Gomez , N. Kapoor , et al., “MatrisomeDB 2.0: 2023 Updates to the ECM‐Protein Knowledge Database,” Nucleic Acids Research 51, no. D1 (2023): D1519–D1530, 10.1093/nar/gkac1009. PMC9825471 36399478

