# Supplementary material for: A Spatiotemporal Single‐Cell Atlas Uncovers Dysregulated ECM Dynamics and Septal Remodeling Arrest in Human Ventricular Septal Defects
Source: Adv Sci (Weinh). 2026 Sep 27:e78016. Online ahead of print. doi: 10.1002/advs.78016 (PMC13616362; doi:10.1002/advs.78016)
Supplement: Supplementary file 1 [file ADVS-9999-e78016-s001.pdf]

Supporting Information

A spatiotemporal single-cell atlas uncovers dysregulated ECM dynamics and septal remodeling arrest in human ventricular septal defects

Xiaoyuan Zhang, Lun Zhu, Ze Sun, Ruolin Jia, Ziming Wang, Jiayao Zhao, Bohao Liu, Haiqing Xiong, Xiaowei Yu*, and Yanzhu Yue**

Xiaoyuan Zhang, Lun Zhu, Ze Sun, Ziming Wang, Jiayao Zhao, Bohao Liu, Yanzhu Yue
Prenatal Diagnosis Center, Reproductive Medicine Center, Department of Cell Fate and Diseases, Jilin Provincial Key Laboratory of Women's Reproductive Health, Jilin Provincial Clinical Research Center for Birth Defect and Rare Disease, the First Hospital of Jilin University, Changchun, Jilin 130061, China
E-mail: yueyanzhu@jlu.edu.cn

Ruolin Jia, Xiaowei Yu
Prenatal Diagnosis Center, Reproductive Medicine Center, the First Hospital of Jilin University, Changchun, Jilin 130061, China
E-mail: yuxiaow@jlu.edu.cn

Haiqing Xiong
State Key Laboratory of Experimental Hematology, National Clinical Research Center for Blood Diseases, Haihe Laboratory of Cell Ecosystem, Institute of Hematology & Blood Diseases Hospital, Chinese Academy of Medical Sciences & Peking Union Medical College, Tianjin 300020, China; Tianjin Institutes of Health Science, Tianjin 301600, China.
E-mail: xionghaiqing@ihcams.ac.cn

* Corresponding Authors: Yanzhu Yue, Xiaowei Yu and Haiqing Xiong

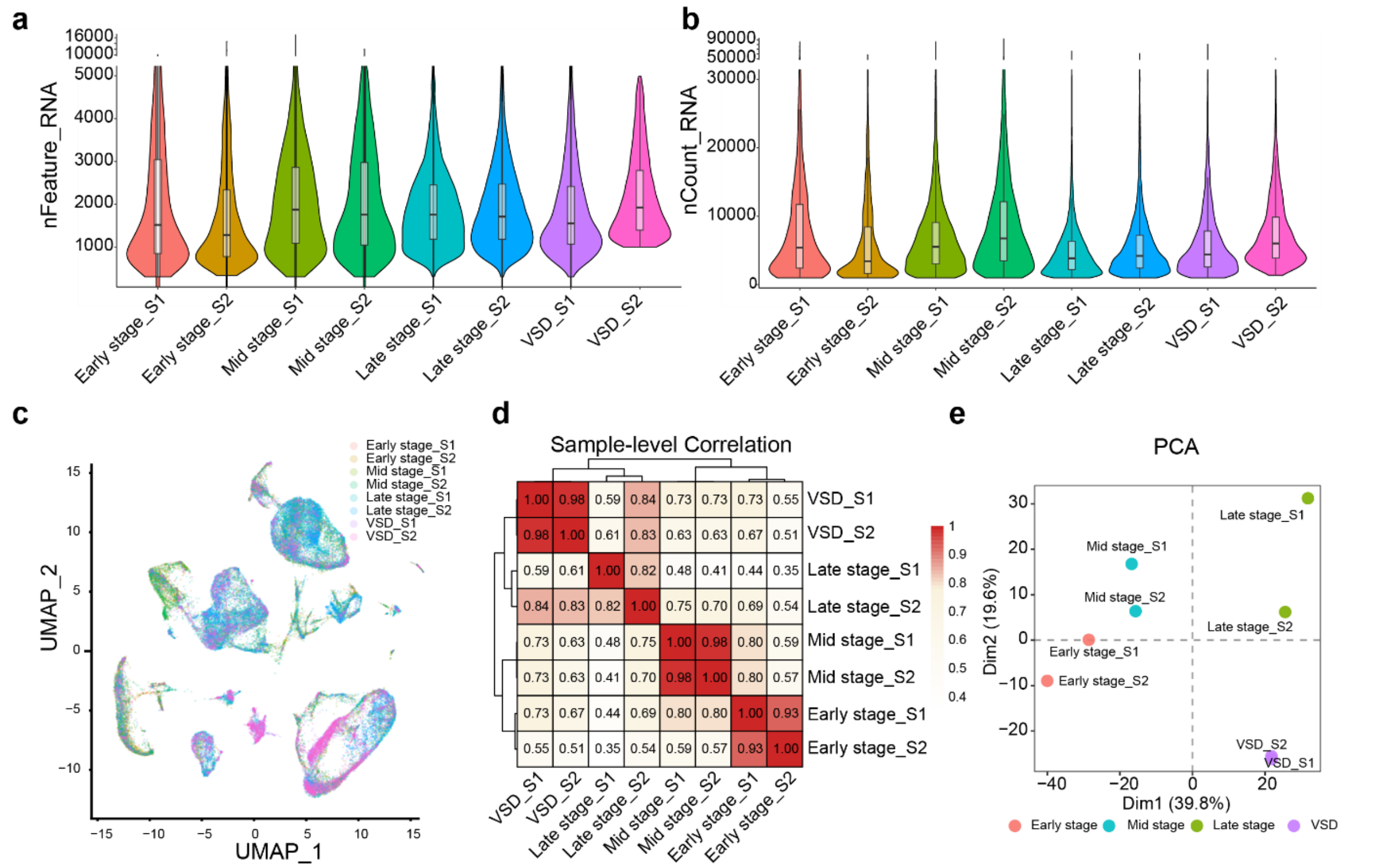

Figure S1. Quality control metrics and batch effect correction.

a,b, Violin plots visualizing the distribution of quality control metrics across all eight biological replicates. Panels show the number of detected genes (**a**) and total unique molecular identifier (UMIs) (**b**) per cell.

c, Global UMAP embedding colored by sample identity demonstrates the effective integration and mixing of cells derived from different biological replicates, ruling out significant batch effects across the dataset.

d, Pearson correlation of pseudobulk expression profiles across all samples. The color of heatmap indicates the magnitude of correlation coefficients, demonstrating high biological reproducibility within groups and robust consistency across replicates.

e, PCA analysis based on pseudobulk expression profiles, clearly captures the clustering of samples within groups and the separation between different groups.

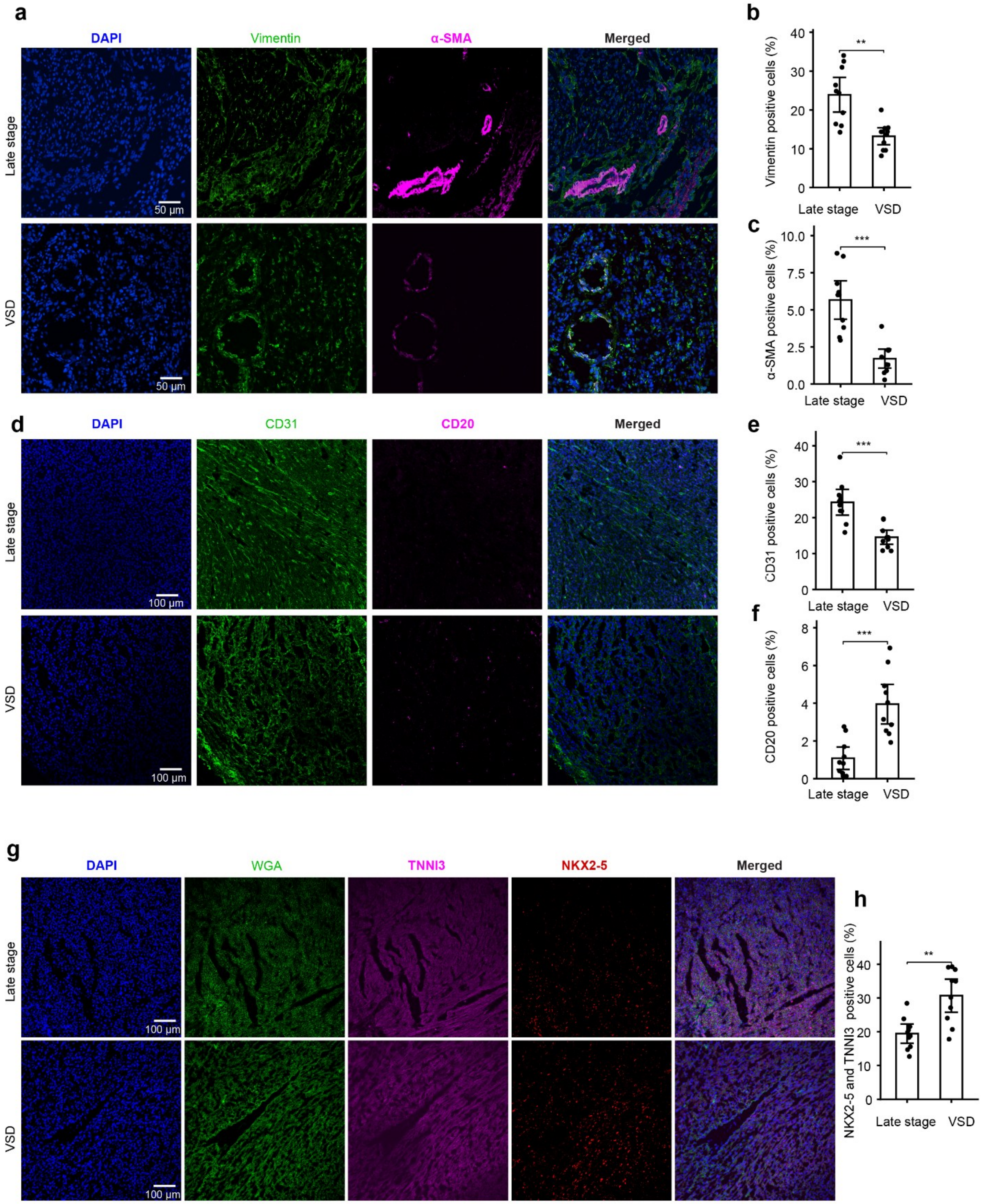

Figure S2. Tissue immunostaining confirms non-myocyte depletion and myocardial expansion in VSD.

a, Representative immunofluorescence images of human fetal septal tissues from Late stage control and VSD hearts, showing *in situ* expression of the stromal markers Vimentin (green) and α-SMA (magenta). Nuclei are counterstained with DAPI (blue). Scale bars, 50 μm.

b,c, Quantitative evaluation of Vimentin-positive fibroblasts and α-SMA-positive smooth muscle cells in tissue sections, validating the depletion of non-myocyte structural lineages in VSD septa. Data are presented as mean ± 95% CI ($n = 2$ biological replicates per group, with 5 randomly selected microscopic fields per sample, totaling 10 fields per group; $^{**}P < 0.01$, and $^{***}P < 0.001$ by two-sided *Student's t-test*).

d, Representative immunofluorescence images of endothelial (CD31, green) and B-cell (CD20, magenta) markers in control versus VSD sections. Nuclei are counterstained with DAPI (blue). Scale bars, 100 μm.

e,f, Quantitative validation showing significant CD31-positive endothelial cell depletion alongside CD20-positive B-cell expansion in control versus VSD sections. Data are presented as mean ± 95% CI ($n = 2$ biological replicates per group, with 5 randomly selected microscopic fields per sample, totaling 10 fields per group; $^{***}P < 0.001$ by two-sided *Student's t-test*).

g,h, Co-immunofluorescence staining for cardiomyocyte lineage markers NKX2-5 (red) and TNNI3 (magenta) merged with WGA cell border staining (green) in Late stage and VSD sections (**g**), alongside cell proportion quantification (**h**) validating VSD myocardial expansion. Data are presented as mean ± 95% CI ($n = 2$ biological replicates per group, with 5 randomly selected microscopic fields per sample, totaling 10 fields per group; $^{**}P < 0.01$ by two-sided *Student's t-test*). Scale bars, 100 μm.

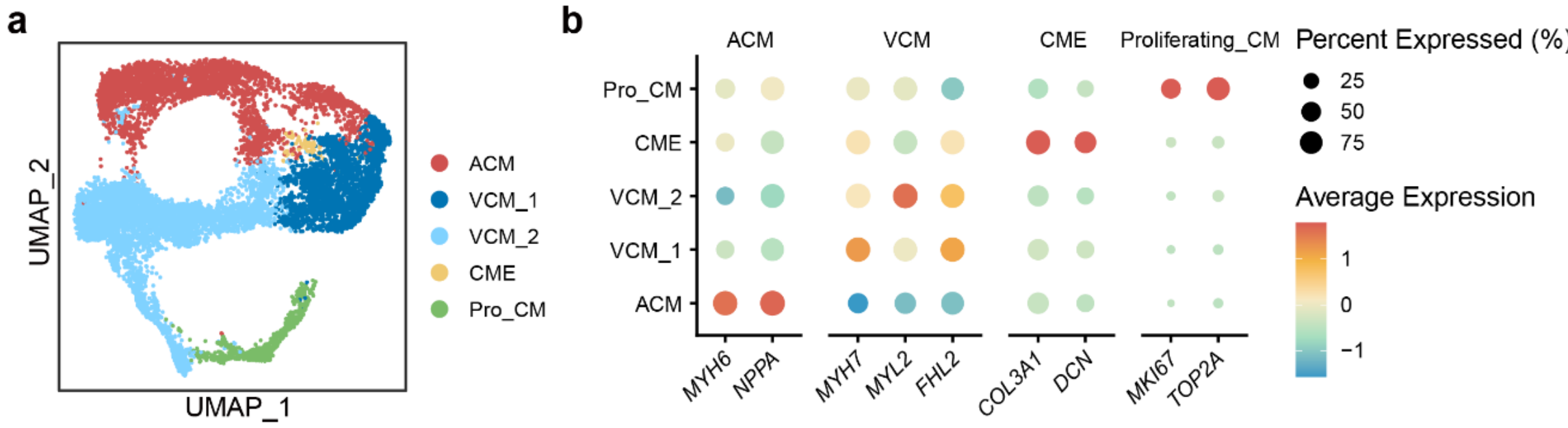

Figure S3. Molecular characterization of cardiomyocyte subpopulations.

a, UMAP embedding showing the sub-clustering of the cardiomyocyte lineage into five distinct phenotypes: Atrial CM (ACM), Ventricular CM subtypes (VCM_1, VCM_2), ECM-enriched CM (CME), and Proliferating CM (Pro_CM).

b, Dot plot visualizing the expression of canonical marker genes used to annotate each subtype. Markers include *MYH6* and *NPPA* for atrial identity; *MYH7*, *MYL2*, and *FHL2* for ventricular identity; *COL3A1* and *DCN* for the ECM-enriched state; and *MKI67* and *TOP2A* for cycling cells. Dot size represents the fraction of expressing cells, and color intensity indicates mean scaled expression levels.

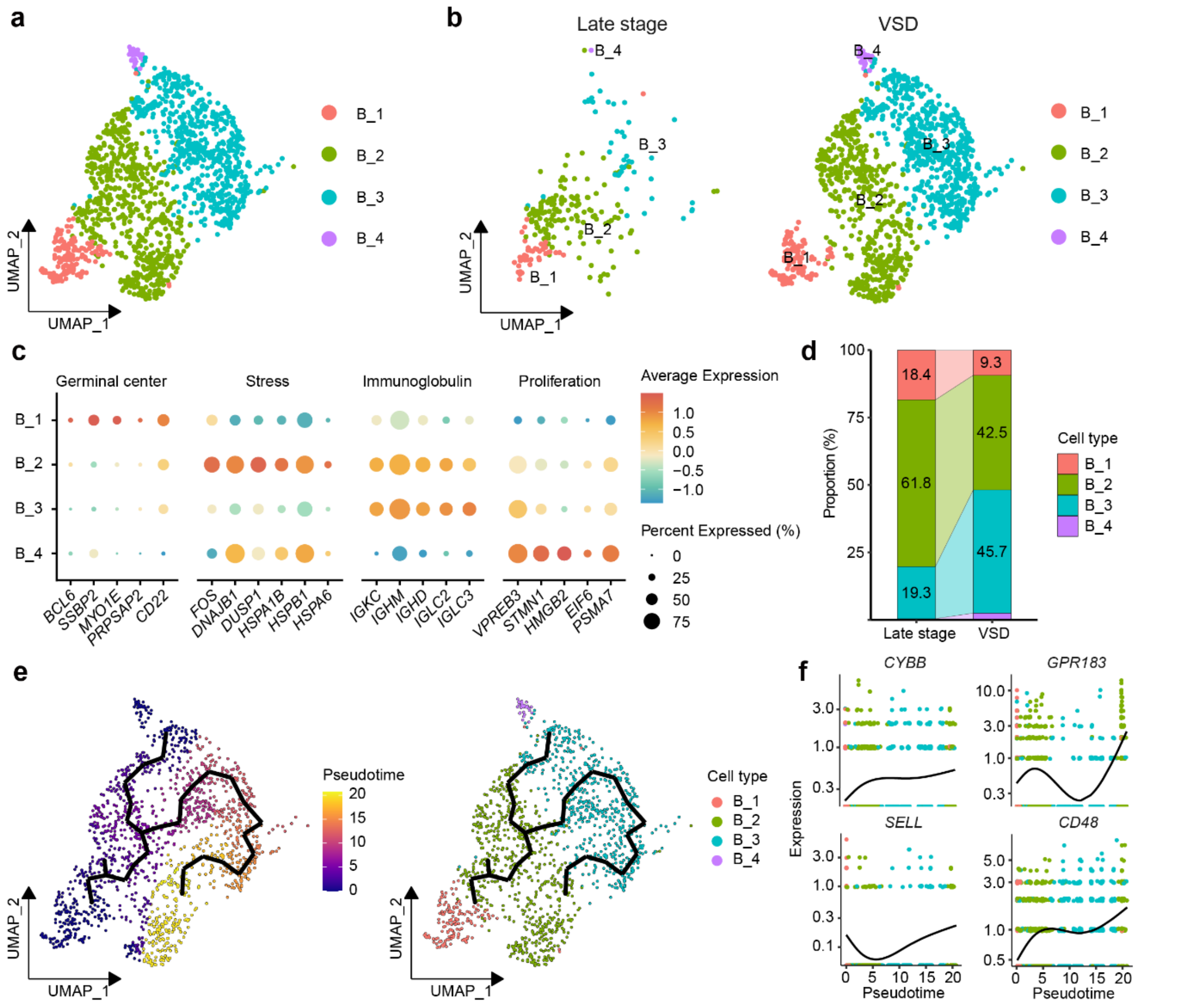

Figure S4. Sub-lineage characterization and inflammatory trajectory of B cells.

a,b, UMAP projections visualize the sub-clustering of the B cell lineage, identifying four distinct subtypes (B_1–B_4) and displaying their distribution across Late stage (**b**, left panel) and VSD (**b**, right panel) samples.

c, Dot plots characterize the functional identity of B cell subtypes based on the expression of canonical markers associated with germinal center formation, stress response, immunoglobulin production, and proliferation.

d, Bar charts quantify the cellular composition shifts, revealing alterations in B cell subtype proportions (notably the expansion of the Immunoglobulin-producing B_3 cluster) in VSD hearts compared to Late-stage controls.

e, Pseudotime trajectory analysis reconstructs the differentiation dynamics, tracing the lineage progression and inferred developmental time (left) mapped onto B cell subtypes (right).

f, Temporal expression profiles of key inflammatory mediators (for example, *CYBB* and *GPR183*) plotted along the pseudotime axis, highlighting the upregulation of immune signaling factors during B cell differentiation.

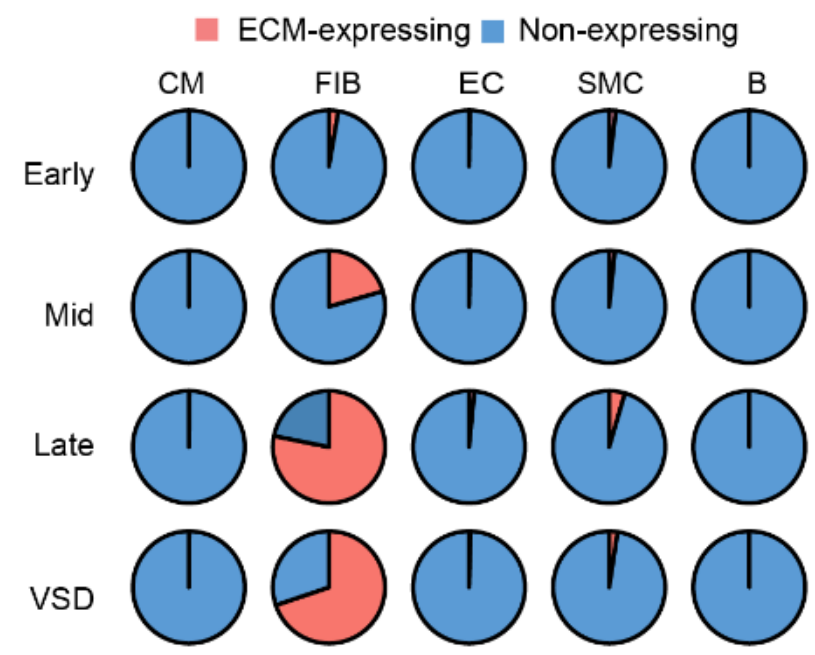

Figure S5. Prevalence of ECM gene expression across cardiac lineages.

Pie charts illustrating the proportion of cells expressing ECM-related genes (ECM-expressing, red) versus non-expressing cells (Non-expressing, blue) within each major cell lineage. Charts are stratified by developmental stage (Early, Mid, Late) and pathology (VSD), highlighting the dominant role of fibroblasts (FIB) in matrix production.

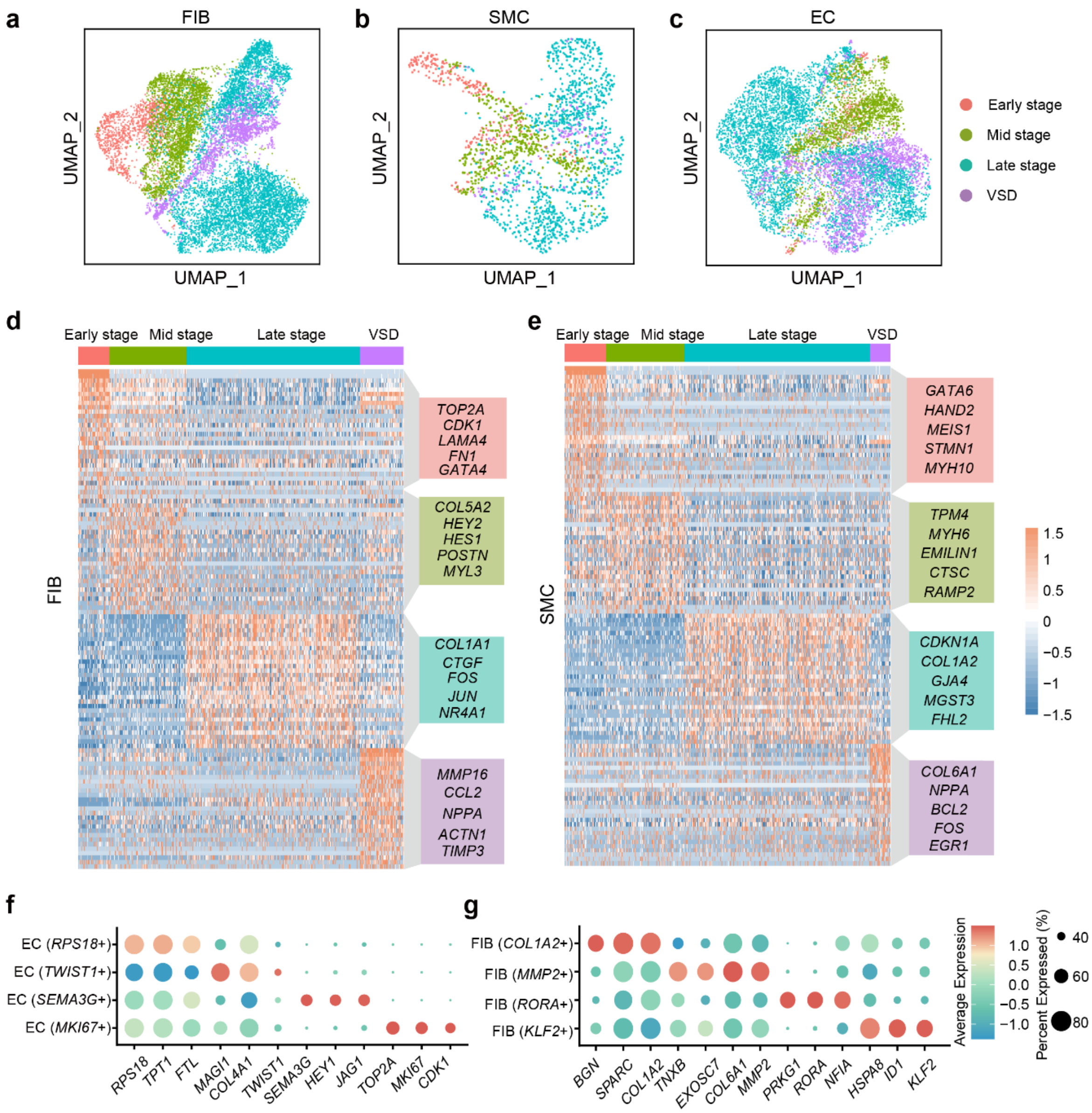

Figure S6. Transcriptional trajectories and pathological signatures of non-cardiomyocyte lineages.

a–c, UMAP projections visualize the continuous developmental landscapes and pathological distributions of major non-cardiomyocyte lineages, including fibroblasts (FIB) (**a**), smooth muscle cells (SMC) (**b**), and endothelial cells (EC) (**c**), colored by sample identity to distinguish cells from early, mid, and late developmental stages versus VSD samples.

d,e, Heatmaps characterizing transcriptional dynamics display the expression of top differentially expressed genes across healthy developmental stages and VSD for fibroblasts (FIB) (**d**) and smooth

muscle cells (SMC) (**e**). The analysis reveals dynamic gene expression shifts during physiological maturation and identifies distinct molecular profiles characterizing the VSD state.

f,g, Molecular characterization of identified subtypes visualized by dot plots, defining distinct transcriptional signatures for endothelial populations (**f**, including *TWIST1*$^+$ and *SEMA3G*$^+$ subsets) and fibroblast populations (**g**, including *MMP2*$^+$ and *COL1A2*$^+$ subsets).

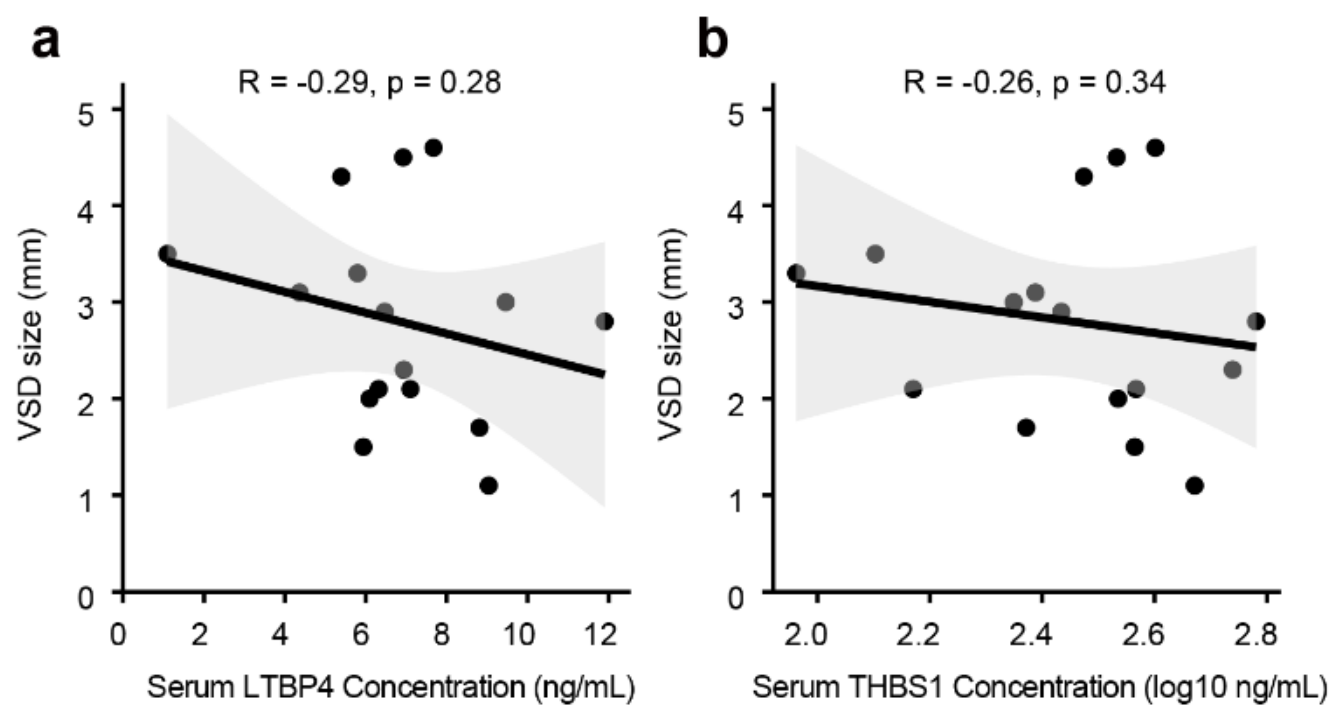

Figure S7. Correlation analysis between maternal serum LTBP4/THBS1 levels and VSD size. a,b, Scatter plots analyzing the relationship between maternal serum concentrations of LTBP4 (**a**) or THBS1 (**b**) and the fetal VSD diameter (n = 16). Spearman correlation coefficients (R) and P-values indicate no statistically significant association.

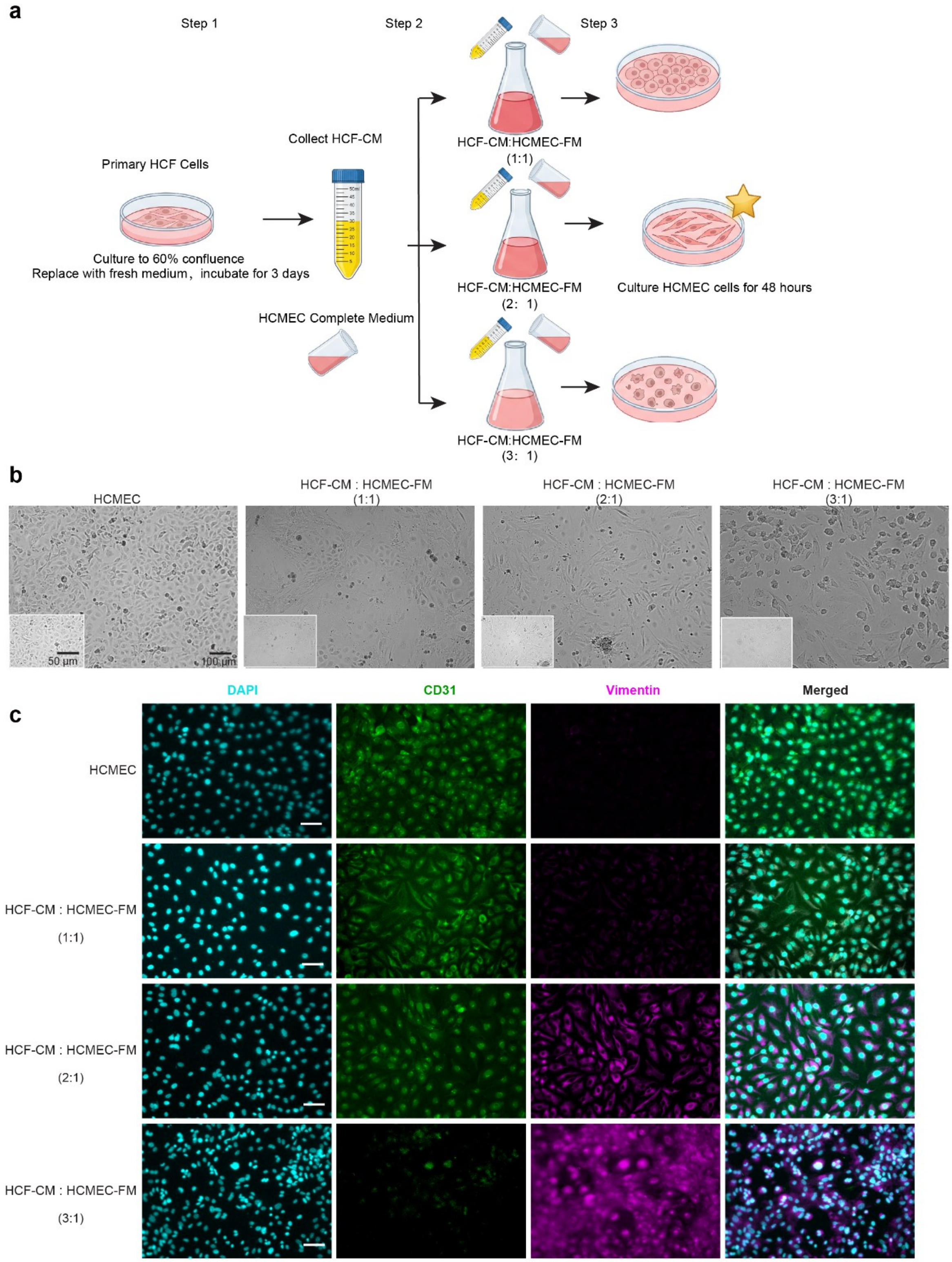

Figure S8. Cardiac fibroblasts induce endothelial-to-mesenchymal transition in microvascular endothelial cells via paracrine signaling.

a, Schematic experimental design validating the functional crosstalk. Human cardiac fibroblast-conditioned medium (HCF-CM) was collected and diluted with fresh endothelial cell growth medium at escalating ratios (1:1, 2:1, and 3:1, HCF-CM: fresh medium). The resulting mixtures were then applied to human cardiac microvascular endothelial cells (HCMECs) for 48 h to evaluate paracrine-induced cell fate transition.

b, Bright-field images reveal a dose-dependent transition of HCMECs from cobblestone endothelial monolayers to spindle-shaped mesenchymal cells upon exposure to HCF-CM mixtures, though at the highest ratio (3:1) the cultures showed overt toxicity, with shrunken cells, widespread detachment, and floating debris. Scale bars, 100 µm and 50 µm.

c, Representative immunofluorescence images of HCMECs across co-culture ratios, demonstrating a progressive loss of the endothelial junction marker CD31 (green) concomitant with the acquisition and intensification of the mesenchymal filament marker Vimentin (magenta). Nuclei are counterstained with DAPI (cyan). Scale bars, 50 µm.

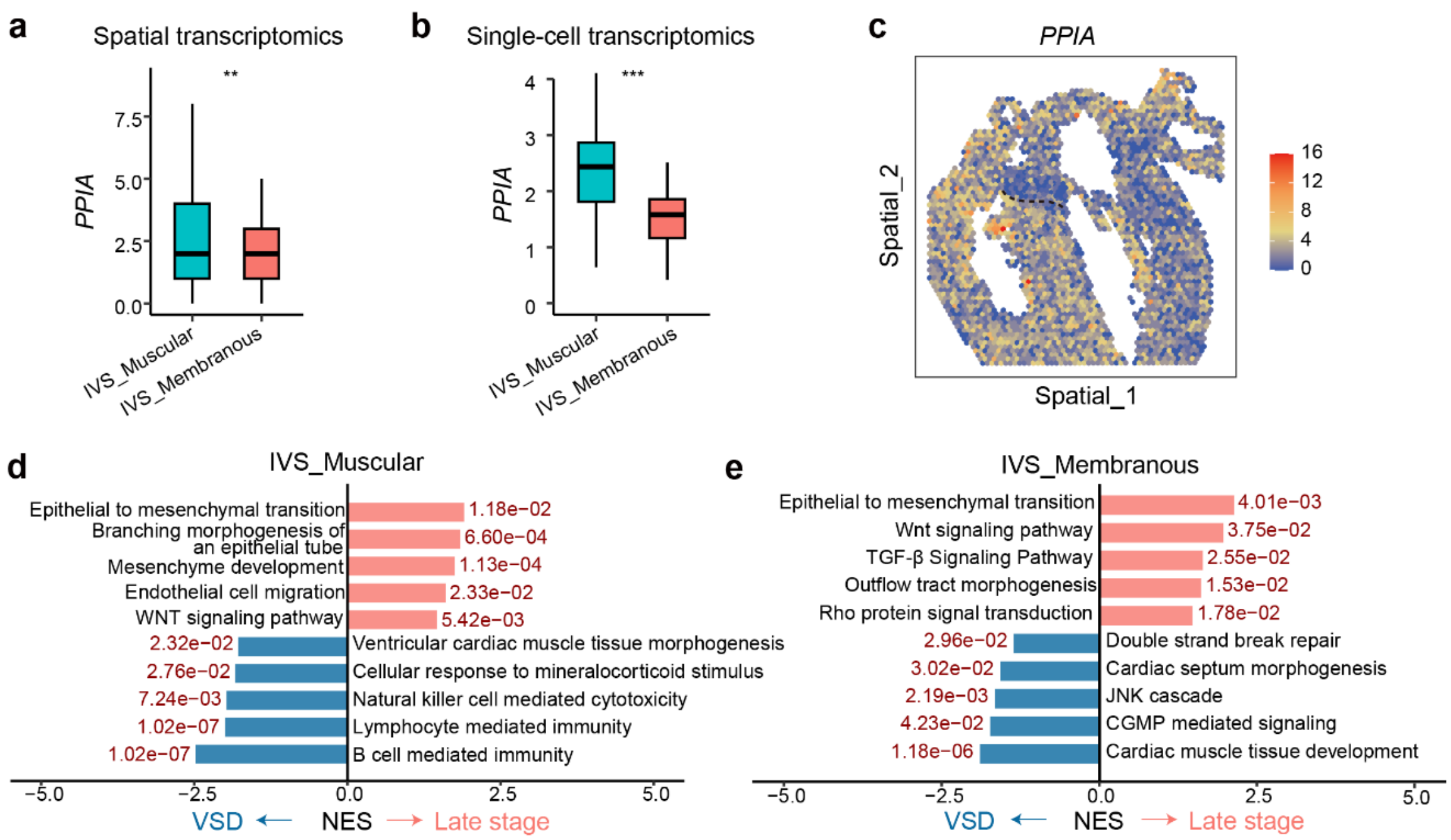

Figure S9. Validation of spatially-resolved single-cell mapping in the muscular and membranous ventricular septum.

a,b, Box plot comparing the region-specific expression levels of *PPIA* in the muscular versus membranous parts of the interventricular septum (IVS), utilizing data from Spatial transcriptomics (**a**) and Single-cell transcriptomics (**b**). (IVS_Muscular: n = 3,524; IVS_Membranous: n = 278. **$P < 0.01$, ***$P < 0.001$ by two-sided *Wilcoxon tests*).

c, Spatial transcriptomic maps illustrating the expression of *PPIA* across the interventricular septum. Expression levels are overlaid on the tissue architecture, highlighting their anatomical localization in the muscular regions.

d,e, Gene Set Enrichment Analysis (GSEA) identifying functional pathways enriched in Late stage (red) versus VSD (blue) samples within the IVS_Muscular (**d**) and IVS_Membranous (**e**). Positive NES indicates enrichment in Late stage, while negative NES indicates enrichment in VSD (| NES | > 1, $P < 0.05$, FDR < 0.25).
